# Molecular architecture of mesoderm cells across early to middle stage of human embryo development at single-cell resolution

**DOI:** 10.1186/s12860-025-00561-9

**Published:** 2025-12-25

**Authors:** Wei Zhang, Haiyan Yu, Zhibin Zhang, Dandan Li, Yane Yang, Wei Shi, Wenting Li, Yan Jiang, Wenlong Hu, Zhipeng Zeng, Xinqiong Liu, Zhanye Zheng, Minglin Ou, Donge Tang, Yong Dai

**Affiliations:** 1 Department of Clinical Laboratory, Peking University Shenzhen Hospital, Shenzhen, China; 2 Department of Experimental Research, South China Hospital, Medical School, Shenzhen University, Shenzhen, China; 3 Pingshan District Peoples’ Hospital of Shenzhen, Shenzhen, China; 4 GCP, Shenzhen Third People’s Hospital, Shenzhen, China; 5 Experimental Center, Shenzhen Pingle Orthopedic Hospital (Shenzhen Pingshan Traditional Chinese Medicine Hospital), Shenzhen, China; 6 Shenzen Far East Women & Children Hospital, Shenzhen, China; 7 Department of Obstetrics and Gynecology, The Second Clinical Medical College of Jinan University, Shenzhen People’s Hospital, Shenzhen, China; 8 Singleron Biotechnologies, Nanjing, China; 9 Clinical Medical Research Center, The Second Clinical Medical College of Jinan University, Shenzhen People’s Hospital, Shenzhen, China; 10 The Second Clinical Medical College of Jinan University, Shenzhen People’s Hospital, Shenzhen, China; 11 Central Laboratory, The Second Affiliated Hospital of Guilin Medical University, Guilin, China; 12 The First Affiliated Hospital, School of Medicine, Anhui University of Science and Technology, Huainan, China

**Keywords:** Mesoderm cell, Embryo development, Organogenesis, Single-cell RNA-seq, Single-cell assay for transposase-accessible chromatin

## Abstract

**Background:**

The differentiation of mesodermal cells (MCs) in the early stage of embryonic development contributes to the organogenesis of several core organs. However, the single-cell molecular architecture of MCs and the key molecular events during the differentiation remain unclear.

**Methods:**

We performed single-cell RNA sequencing (RNA sequencing) and single-cell assay for transposase-accessible chromatin (ATAC-Seq) to analyze the developmental features of MCs to heart, kidney, spleen, liver, and brain in human embryos at gestational ages 7–17 weeks.

**Results:**

We found that *EGR1* might be relevant to the differentiation of heterogeneous MC sub-clusters. Meanwhile, *RPL10P9*^*+*^*PTMAP5*^*+*^ MCs had the closest expression profiling with endocardial cells. *NDUFA4L2*^*+*^*A2M*^*+*^ MCs presented the potentials to form endothelial cells (ECs) and hematopoietic stem cells, and *MEF2C* might be involved in this process.

**Conclusions:**

These findings provide insights into the molecular architecture and lineage progression of MCs during early human embryonic organogenesis, offering a valuable reference for regenerative medicine and organ bioengineering.

**Supplementary Information:**

The online version contains supplementary material available at 10.1186/s12860-025-00561-9.

## Introduction

During the early stages of mammalian embryonic development, the three germ layers - endoderm, mesoderm, and ectoderm - differentiate into downstream stem and progenitor cells [1, 2]. This period is critical because it establishes cell fate decisions and initiates the organogenesis of several major organs. Constructing a reference atlas of human cell types based on developing tissues would provide a fundamental framework for systematically understanding how molecular and cellular events contribute to both rare and common developmental disorders. Although several studies have characterized organ-specific features at mid-gestational stages [3], systematic investigations covering the developmental transition from early to mid-gestation remain limited.

Human samples in this window (gestational weeks GW 7–17, approximately corresponding to embryonic days 8.5–14.5 in mice) are particularly valuable [4], as mesodermal cells (MCs) undergo lineage specification into multiple downstream cell types, including endothelial and hematopoietic cells [1, 2]. This process is fundamental for organogenesis, yet remains poorly defined in humans due to the scarcity of embryonic materials.

Many degenerative diseases originate in mesoderm-derived organs, such as muscular dystrophies and chronic heart diseases [5]. In recent years, stem cell therapy has attracted significant attention in the field of organ bioengineering [6]. Single-cell technologies have provided powerful tools to resolve cellular heterogeneity and trace lineage trajectories, thereby offering an important theoretical basis for stem cell therapy [7, 8]. Together, these advances create an unprecedented opportunity to chart mesodermal development at single-cell resolution and uncover molecular regulators relevant for regenerative medicine.

In this study, we systematically analyzed the heterogeneity and developmental trajectories of MCs from human embryos using single-cell RNA sequencing (scRNA-seq) and single-cell assay for transposase-accessible chromatin sequencing (scATAC-seq). Specifically, we characterized the molecular events underlying the differentiation of MCs into endocardial cells, ECs, and hematopoietic lineage cells within human fetal brain, heart, liver, kidney, and spleen. Furthermore, we identified putative regulatory factors involved in these differentiation processes. Collectively, our work delineates the functional roles, molecular events, and developmental trajectories of distinct MC sub-clusters (subnets of MC cells), providing a framework for future in-depth studies of early mesodermal development and offering molecular insights relevant to organ repair and regeneration.

## Materials and methods

### Sample collection

Our study was approved by the Ethics Committee of Shenzhen People’s Hospital (LL-KY-2019591). All the participants have reviewed the purpose and procedures of this study and were volunteer to participate in the project and signed the informed consent. The embryos used in this study were obtained from elective terminations of otherwise healthy pregnancies, collected as part of routine clinical practice. The samples were generally collected within 1–2 h after expulsion to ensure their freshness and integrity.

The human embryos of different stages (GW07, GW08, GW10, GW11, and GW17) were collected and then cleaned with ice-cold saline solution. The size and morphology of the embryos were recorded using a camera, and each organ was collected in a biosafety cabinet, including hearts, brains, livers, and kidneys. Subsequently, the organs were washed with saline solution for three times. Next, each sample was cut into small portions (5 × 5 × 5 mm^3^) on ice using sterile scissors, and four pieces were then stored in GEXSCOPETM Tissue Preservation Solution (Singleron Biotechnologies) for the following experiments. The whole process was completed within 1 h.

### Tissue dissociation

The tissues in GEXSCOPETM Tissue Preservation Solution were then washed with Hanks Balanced Salt Solution (HBSS) for three times and minced into smaller pieces (1–2 mm). Next, the tissues were incubated in 2 mL GEXSCOPETM Tissue Dissociation Solution (Singleron Biotechnologies) at 37℃ for 15 min with sustained agitation. Subsequently, the mixture was filtered using a 40-micron sterile strainer (Corning) to obtain single cells. The single-cell solution was then centrifuged at 400 × g for 5 minutes. Then the supernatant was discarded, and the sediment was resuspended in 1 mL PBS (HyClone). 2 mL GEXSCOPETM red blood cell lysis buffer (Singleron Biotechnologies) was added into the resuspended cells and incubated at 25 °C for 10 min to remove the red blood cells. The mixture was then centrifuged at 500 × g for 5 min. The supernatant was discarded, and the residual cells were resuspended in 1 mL PBS. Finally, the concentration and viability of cells were calculated using the TC20 automated cell counter (Bio-Rad). The cell concentration was adjusted into 1 × 10^5^ cells/mL using PBS for library construction.

### Library preparation

The scRNA-seq libraries were constructed according to the instructions (Singleron Biotechnologies). The libraries were sequenced using a sequencer (Illumina Nova6000) with 150 bp paired-end reads. The libraries for scATAC-seq were prepared using a Chromium Single Cell ATAC Reagent Kit according to the manufacturer’s instructions (10 X Genomics CG000168 Rev. B). Briefly, the ATAC Buffer and ATAC Enzyme were added into the nuclei following incubating at 37 °C for 60 min. Next, the Sample Index PCR Mix and Chromium i7 Sample Index were mixed with the amplification product to obtain the indexed sequencing libraries. Finally, the libraries were sequenced using a sequencer (Illumina Nova6000) with 50 bp paired-end reads.

### Single-cell gene expression matrices

The gene expression matrices were generated from raw sequencing reads using Cell Ranger (v3.0.x–v3.1.0). Briefly, Read 1 sequences lacking poly(T) tails were discarded. The cell barcodes - short DNA sequences attached to each cell’s cDNA or chromatin fragment that enable the identification of reads from individual cells after sequencing — and unique molecular identifiers (UMIs) were then extracted for downstream analysis. Read 2 sequences were aligned to the human reference genome GRCh38 (Ensembl v92) using the STAR aligner (v2.7.3a; https://github.com/alexdobin/STAR) [9] after trimming adapter sequences and poly(A) tails. Reads sharing the same cell barcode, UMI, and gene were grouped to quantify the number of UMIs per gene per cell. Finally, cell counts were determined using the “knee” method implemented in Cell Ranger (v3.0.x–v3.1.0).

### Quality controlling

Ambient RNA contamination was assessed and found to be minimal; therefore, ambient-RNA decontamination was not applied. The quality control (QC) and dimensionality reduction were processed using Seurat v3.1.2^10^. Here, “doublets” denote two or more cells captured under a single barcode that yield a composite transcriptome. Putative doublets were flagged when a cell co-expressed mutually exclusive lineage markers from unrelated lineages and/or fell in the top 2% of the per-cell distribution for total UMI counts per cell (nUMI) or number of detected genes per cell (nGene); flagged cells were removed prior to downstream analyses. The cells were filtered following the criteria: 1, cells with gene count less than 200 were excluded; 2, cells with gene count ranked in the top 2% were excluded; 3, cells with UMI count ranked in the top 2% were excluded; 4, cells with 50% of their genes from mitochondria were excluded. The genes were filtered following the criteria: 1, genes expressed in less than five cells were excluded. Ultimately, 73,949 cells, 1219 genes, and 3807 UMIs per cell on average were obtained for the following investigation.

### Clustering

Next, Seurat v3.1.2 was utilized to normalize gene expression using the functions named NormalizeData and ScaleData [10]. Subsequently, the top 2000 variable genes were selected using the FindVariable function for Principal Component Analysis (PCA). Cells were separated into clusters by FindClusters(), using the top 20 principle components and resolution parameter at 2.0. For sub-clustering of specific cell types (e.g., MCs), the resolution was set to 1.2. The Uniform Manifold Approximation and Projection (UMAP) algorithm was then applied to visualize cells in a two-dimensional space.

### Differentially-expressed genes (DEGs)

Genes expressed in more than 10% of the cells of one cluster, simultaneously with an average LogFold Chang (LogFC) greater than 0.25, were regarded as the DEGs. The function FindMarkers of Seurat v3.1.2 was performed to screen for the DEGs [10], using the Wilcoxon rank-sum test as the default statistical method with p-values adjusted by the Benjamini–Hochberg false discovery rate (FDR) correction.

### Cell type annotation

The biomarkers of cells were selected out following the criteria: 1, canonical gene markers; 2, being up-regulated in the cells versus other cells. Subsequently, the cell type of each cluster was annotated with the biomarkers using SynEcoSys database (Singleron Biotechnologies, https://www.synecosys.com) [11]. The dot plots displaying the expression of biomarkers were depicted with the function DotPlot of Seurat v3.1.2 [10]. To assign one of the six major cell types to each cluster, each cluster were scored with the normalized expression of the following gene markers: hematopoietic cells (*PTPRC*, *CD3D*, *MS4A1*, *LYZ*, *AHSP*, *GYPA*, *HBA1*), epithelial cells (*EPCAM*, *NPHS2*, *CUBN*, *KRT8*, *CLDN1*), stromal cells (*DCN*, *LUM*, *VIM*, *COL5A1*), myocytes (*TNNI3*, *TNNT2*, *MYH6*, *NPPA*, *TNNC1*), neural cells (*SNAP25*, *DCX*, *SYT1*, *STMN2*), endocrine cells (*CHGA*, *SCG2*, *TH*, *CHGB*). The automated annotation was further cross-validated by manual inspection of canonical marker expression to ensure biological plausibility and consistency across clusters. The annotation was then manually examined. The final results were visualized using UMAP.

### Trajectory analysis

According to the standard method, cellular developmental trajectories were reconstructed using Monocle2 analysis [12]. Monocle2 infers pseudotime by ordering cells along trajectories based on transcriptional similarity, using DDRTree for dimensionality reduction. The top 2000 highly variable genes were selected out using Seurat v3.1.2 [10] and the dimensionality was reduced using DDRTree. To screen out the genes with highly variable expression along with the pseudo-time, the overlapping analysis was performed between 2000 DEGs selected out above, and the genes highlighted using the function differentialGeneTest (q value < 0.001). Subsequently, the intersected genes were clustered into five clusters using the function named plot_pseudo-time_heatmap.

Furthermore, the R package slingshot (1.4.0) was used to model the developmental trajectories after the clustering of neurons. Slingshot infers lineages by constructing a minimum spanning tree of clusters and then fitting smooth curves to represent developmental paths. The SingleCellExperiment objects were created using the single-cell expression matrix and then used as an input for the slingshot analysis.

### Analysis of transcriptional factors (TFs)

The upstream TFs of target genes were annotated and predicted using AnimalTFDB database (http://bioinfo.life.hust.edu.cn/AnimalTFDB4/). The potential active TFs were screened out based on the scRNA expression matrix using the SCENIC R toolkit [13]. SCENIC is a computational framework that integrates co-expression and motif analysis to reconstruct gene regulatory networks and identify cell states from single-cell transcriptomic data. Specifically, SCENIC R toolkit (v1.1.2-3) first infers co-expression modules using GENIE3 (v1.6.0), then refines these modules by assessing cis-regulatory motif enrichment with RcisTarget, and finally quantifies the activity of each regulon at the single-cell level using AUCell (v1.6.0).

Specifically, the R package GENIE3 (v1.6.0) was used to predict a regulatory network based on the co-expression of the regulators and targets. The R package RcisTarget (v1.6.0) was utilized to search for the binding motifs of TFs. Genes involved in the predicted regulatory network were defined as a gene set. The R package AUCell (v1.6.0) was utilized to calculate the AUC value of the gene set to assess the activity of the regulatory network in cells. The RunChromVAR function in the R package chromVAR (v1.8.0) was used to calculate the TF activity on the ATAC-seq data using the tested motif dataset from the HOMER human TF binding models database.

### scATAC-seq data processing and annotation

The scATAC-seq data were aligned to the GRCh38 genome using Cell Ranger ATAC v1.2.0. The adapters, low-quality sequences, and repeat sequences were removed after quantification. The cell types with cell number more than 50 were selected out for the following analysis. Peak calling and peak merge were performed using MACS2 (v2.1.1) [14]. The default threshold of q value was set as 0.05. MACS2 identifies regions of open chromatin by modeling background noise and detecting significant enrichment of ATAC-seq reads. Peaks located at gene functional regions were calculated using bedtools. The reads were displayed using Integrative Genomics Viewer (IGV, v2.4.x; https://software.broadinstitute.org/software/igv/).

The differentially accessible peaks were identified using SnapATAC (Single-Nucleus Analysis Pipeline for ATAC-seq, v1.0.0, https://github.com/r3fang/SnapATAC) [15]. The peaks with LogFC > 0 were selected out, and the top 200 (p value) peaks were used for the following analysis. SnapATAC was utilized to find the specific peaks of each type of cells. The motifs were obtained using the script findMotifsGenome.pl of Homer (v4.11) based on specific peaks, and the script annotatePeaks.pl was used to annotate the peaks [16]. HOMER performs motif enrichment analysis by scanning peak sequences for known TF binding motifs and evaluating their overrepresentation. The motifs (p value < 0.05) of each type of cells were depicted using the statistical graph.

### Gene activity analysis

A cell-by-bin count matrix at 5-kb resolution was generated from the aligned ATAC-seq fragments. The resulting bin matrix was then incorporated into the Snap object using the function addBmatToSnap in SnapATAC (v1.0.0) for downstream analysis [15]. Gene activity scores were then inferred using createGmatFromMat to build a cell-by-gene matrix, which links chromatin accessibility at regulatory elements to putative target genes. These scores were visualized at single-cell resolution by UMAP with plotFeatureSingle.

### Cell differentiation potential evaluation

CytoTRACE (v0.3.3) [17] was applied to estimate the developmental potential of each sub-cluster. This algorithm infers cellular differentiation states from scRNA-seq data by leveraging the number of genes detected per cell together with their expression profiles. In practice, cells with broader transcriptional diversity are predicted to be less differentiated, whereas those with restricted gene expression are inferred to be more lineage-committed. Using this approach, we obtained a quantitative ranking of differentiation potential across the identified cell clusters.

### Single-cell entropy analysis

To assess cellular stemness, we applied SLICE (v0.99.0), a computational framework that quantifies the entropy of gene expression at the single-cell level [18]. In brief, SLICE infers the differentiation potential of cells by measuring transcriptional entropy, under the rationale that undifferentiated cells generally exhibit greater transcriptional diversity compared to lineage-committed cells. Prior to analysis, ribosomal genes were excluded to avoid bias in entropy estimation. A SLICE object was then constructed, and the getEntropy function was used to calculate bootstrap-based single-cell entropy values. These entropy scores provided an index of stemness for each cell, enabling us to compare differentiation potential across sub-clusters.

### Pathway enrichment analysis

To gain insight into the potential biological roles of the identified gene sets or cell clusters, we performed Gene Ontology (GO) [19] and Kyoto Encyclopedia of Genes and Genomes (KEGG) [20] pathway enrichment analyses. These analyses were implemented using the clusterProfiler R package (v3.16.1) [21], which provides a comprehensive framework for statistical analysis and visualization of functional profiles of genes and gene clusters. The GO terms were categorized into molecular function (MF), biological process (BP), and cellular component (CC), enabling a systematic interpretation of gene functions from different biological perspectives. KEGG analysis was performed in parallel to highlight canonical signaling pathways and metabolic processes potentially involved in regulating cellular states. Pathways with an adjusted p value (p_adjusted) < 0.05 were considered statistically significant, and the enriched terms were visualized as bar plots to facilitate interpretation.

For Gene Set Variation Analysis (GSVA), we applied a non-parametric, unsupervised method that estimates variation of pathway activity over a population of samples in an expression dataset [22]. Specifically, the average gene expression values of each annotated cell type were used as input, allowing us to infer pathway activity at the single-cell population level rather than relying on individual marker genes. This approach provides a robust means of identifying functional heterogeneity among cell subsets and tracking dynamic changes in pathway activity during development.

### Statistical analysis

DEGs were identified using the Wilcoxon rank-sum test, with p-values adjusted by the FDR correction (Seurat FindMarkers). Differentially accessible chromatin peaks from scATAC-seq were determined using an exact test (SnapATAC). For trajectory analysis (Monocle2 and Slingshot), pseudotime ordering was based on transcriptional similarity, and significantly dynamic genes along pseudotime were detected using the differentialGeneTest function (q < 0.001). In the GeneSwitches analysis, logistic regression was used to model bimodal gene expression states, and model performance was evaluated using McFadden’s pseudo-R². For TF activity inference (SCENIC, chromVAR), motif enrichment significance was assessed with Fisher’s exact test. Pathway enrichment analyses (GO and KEGG) were carried out with the clusterProfiler package, applying Wald tests with multiple-testing correction (p_adjusted < 0.05). For GSVA, a non-parametric, unsupervised method was used to evaluate pathway activity across cell populations. Jaccard similarity coefficients were calculated to quantify the overlap between DEGs and cell type marker genes. Unless otherwise specified, all statistical tests were two-sided, and results with adjusted p-values < 0.05 were considered statistically significant.

### Summary of software, databases, and tools

To facilitate reproducibility and clarity, all software, analytical methods, and databases used in this study are summarized in Table S1.

## Results

### Integrative scRNA-seq and scATAC-seq analysis reveals cellular heterogeneity across embryonic organs

**Fig. 1:**
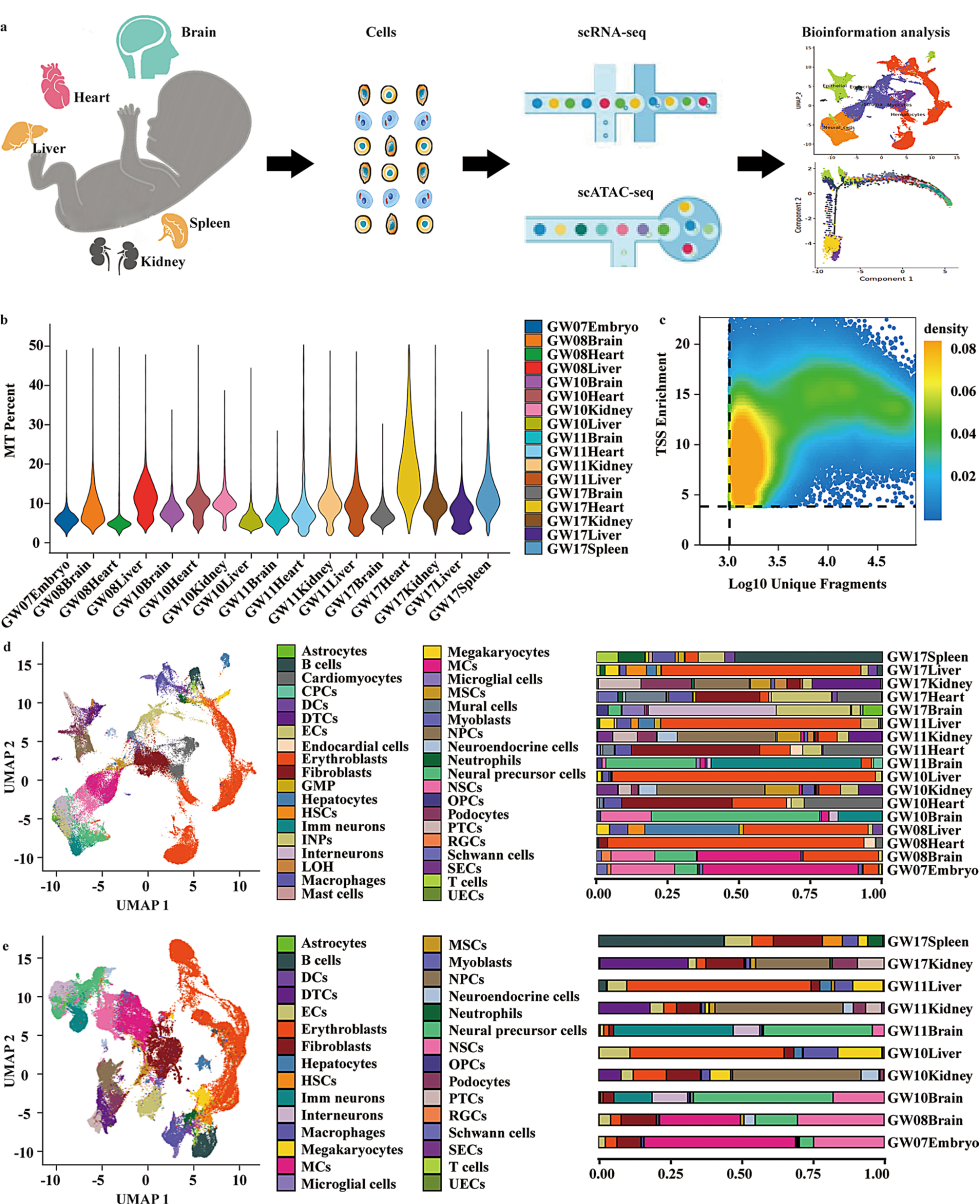
The single-cell profiling of fetal organs detected using scRNA-seq and scATAC -seq. **a**, The workflow of single-cell sequencing and analysis. **b**, Quality control metrics for single-cell transcriptomes across all samples, showing mitochondrial gene percentage per sample at different stages of gestation across different tissues. MT percentage indicates mitochondrial gene percentage. **c**, Quality assessment of scATAC-seq libraries. Scatter density plot showing transcription start site (TSS) enrichment versus the number of unique fragments per cell (log10 scale). Most cells displayed high fragment counts and strong TSS enrichment, indicative of good signal-to-noise ratios and overall high-quality chromatin accessibility profiles. Dashed lines mark standard quality thresholds used to filter low-quality cells. **d**, Single-cell transcriptomic landscape of five fetal organs (brain, heart, liver, kidney, spleen) and a 7-week entire embryo. The bar plot on the right shows the relative abundance of each cell type across developmental stages (GW07–17) and organs (embryo, brain, heart, liver, kidney, spleen). **e**, Single-cell open chromatin profiles of five fetal organs (brain, heart, liver, kidney, spleen) and a 7-week entire embryo. The corresponding bar plot indicates distribution patterns across tissues and gestational weeks. CPCs, Choroid plexus cells; DCs, Dendritic cells; DTCs, Distal tubule cells; ECs, Endothelial cells; GMP, Granulocytes macrophages progenitor cells; HSCs, Haematopoietic stem cells; Imm neurons, Immature neurons; INPs, Intermediate neural progenitors; LOH, Loop of Henle cells; MCs, Mesoderm cells; MSCs, Mesenchymal stem cells; NPCs, Nephron progenitor cells; NSCs, Neural stem cells; OPCs, Oligodendrocyte precursor cells; PTCs, Proximal tubule cells; RGCs, Retinal ganglion cells; SECs, Steroid endocrine cells; UECs, Ureter epithelial cells

In the study, to explore the heterogeneity of MCs and the key factors for downstream differentiation, we collected hearts, kidneys, livers, spleens, and brains of GW08 to GW17 embryos, performed scRNA-seq and scATAC-seq, and analyzed MC heterogeneity and differentiation (Fig. 1a, Table S2). scRNA-seq captures transcriptional states at single-cell resolution, whereas scATAC-seq profiles chromatin accessibility to reveal regulatory landscapes. Integrating these modalities provides complementary insights into both gene expression and upstream regulation, thereby enabling robust cell-type identification and regulatory inference.

We first performed QC for both datasets. For scRNA-seq, most cells showed robust transcript detection with a median of 500–2000 genes per cell and low mitochondrial gene proportions (MT %), suggesting high RNA integrity and minimal stress artifacts (Fig. 1b, Fig. S1a). For scATAC-seq, cells displayed strong transcription start site (TSS) enrichment and sufficient fragment counts (Fig. 1c), demonstrating a clear signal-to-noise ratio and reliable chromatin accessibility profiles. Together, these QC metrics confirmed that both datasets met established single-cell quality standards, providing a solid foundation for subsequent clustering, trajectory inference, and regulatory network reconstruction. Median gene counts (for scRNA-seq) and median fragment counts (for scATAC-seq) across all samples are summarized in Table S3, reflecting overall sequencing depth and data quality.

We initially grouped all clusters into six major cellular compartments, including endocrine cells, myocytes, epithelial cells, neural cells, stromal cells, and hematocytes (Fig. S1b). UMAP visualization of scRNA-Seq data further revealed clear segregation of major cell types, including immune lineages (B cells, T cells, macrophages, dendritic cells), endothelial cell (EC) sub-clusters (embryo/brain/heart/kidney/liver/spleen ECs), mesenchymal cells, and diverse tissue-specific populations such as cardiomyocytes, hepatocytes, and nephron progenitor cells (NPCs) (Fig. 1d, Table S4-S5a). Notably, we identified a total of 5,888 MCs across all samples, as determined by clustering analysis based on scRNA-seq profiles. Among these, the majority were derived from the 7-week embryo (*n* = 4030) and the 8-week fetal brain (*n* = 1628), as detailed in Table S5a.

To confirm the accuracy of cell type annotation, we examined the expression of canonical marker genes across the identified clusters. Major cell lineages such as hematocytes (*AHSP*,* GYPA*), stromal cells (*COL5A1*,* DCN*), neural cells (*DCX*,* STMN2*), epithelial cells (*KRT8*,* EPCAM*), myocytes (*MYH6*,* TNNT2*), and endocrine cells (*CHGA*,* CHGB*) exhibited distinct marker expression patterns (Fig. S1b-c, Table S4).

We next compared the cellular composition across different tissues and developmental stages. As shown in Fig. S1d, the spleen was dominated by immune cells such as B cells, T cells, and macrophages; the liver by hepatocytes together with endothelial and stromal cells; the kidney by podocytes and tubular epithelial populations; and the heart by cardiomyocytes and endocardial cells. Brain samples were enriched in neural progenitors, neurons, and glial populations. Early embryonic samples (approximately GW07–GW10) contained relatively higher proportions of hematopoietic and stromal cells, whereas neuronal populations became increasingly predominant by GW17.

Consistently, the scATAC-Seq data recapitulated a similar cellular architecture based on chromatin accessibility profiles. Major cell identities inferred from accessible regulatory elements aligned well with scRNA-Seq annotations, and tissue-specific patterns were also preserved (Fig. 1e, Table S5b). Additionally, we integrated scRNA-Seq and scATAC-Seq data using a joint embedding approach. As shown in Fig. S1e, cells from both modalities were well-aligned in the shared UMAP space, demonstrating a high degree of concordance between gene expression and chromatin accessibility at the single-cell level. This integrative analysis validated the robustness of our cell type annotations across multi-omics modalities.

### MCs as progenitors driving multi-organ and hematopoietic lineages

Based on this atlas, developmental trajectory inference was performed to explore lineage relationships. trajectory analysis revealed that MCs serve as common progenitors for multiple organ-specific lineages (Fig. S2a–f).

In the brain, trajectory analysis suggested the predominance of neuroectodermal lineages, with neural stem cells (NSCs) and precursors differentiating into interneurons and astrocytes (Fig. S2a). Monocle2-based trajectory analysis (Fig. S2b) identified dynamic genes enriched for neural markers, including *SOX4*,* DCX*, and *STMN2*, consistent with progressive neural differentiation.

In the heart, MCs were enriched at GW07 but gradually decreased thereafter, concomitant with the expansion of cardiomyocytes, endothelial cells, and fibroblasts (Fig. S2c). Monocle2-based trajectory analysis (Fig. S2d) highlighted cardiac developmental markers such as *NPPA*, reflecting the transition toward specialized cardiac lineages.

In the liver, mesodermal clusters persisted across stages and contributed to both stromal and hematopoietic lineages (Fig. S2e). Trajectory-associated gene analysis (Fig. S2f) demonstrated robust induction of hematopoietic markers, including *PF4* (megakaryocyte/platelet lineage) and *FLT1* (hematopoietic/endothelial progenitors), consistent with the fetal liver’s role as the primary hematopoietic organ.

Together, these results reveal both compositional and transcriptional programs that define organ-specific lineage emergence, and highlight the transient but critical role of MCs in seeding multiple organ and hematopoietic trajectories.

### Single-cell trajectory and regulatory landscape of MCs during early-to-mid embryogenesis

During early embryogenesis, the three germ layers generate progenitor populations that drive organogenesis. Although mid-gestational features have been described [3], systematic analyses of the early-to-mid transition are scarce. Therefore, we focused our subsequent analyses on MCs to delineate their differentiation into multiple organ-specific lineages. In our dataset covering gestational weeks GW7-17, classical early mesodermal markers such as *BRACHYURY (T)*,* EOMES*, and *TBX6* were expressed at very low or undetectable levels, consistent with their well-documented transient expression restricted to the primitive streak and early induction stages [23–25]. This pattern confirms that our samples capture post-gastrulation mesoderm undergoing lineage specification rather than primitive streak formation. Instead, we detected moderate expression of the classical mesodermal markers *PDGFRA* and *MIXL1*, together with robust expression of *FOXC1*,* MEST*, and *MDK*, which served as representative markers of mesodermal populations during organogenesis (Table S4).

**Fig. 2:**
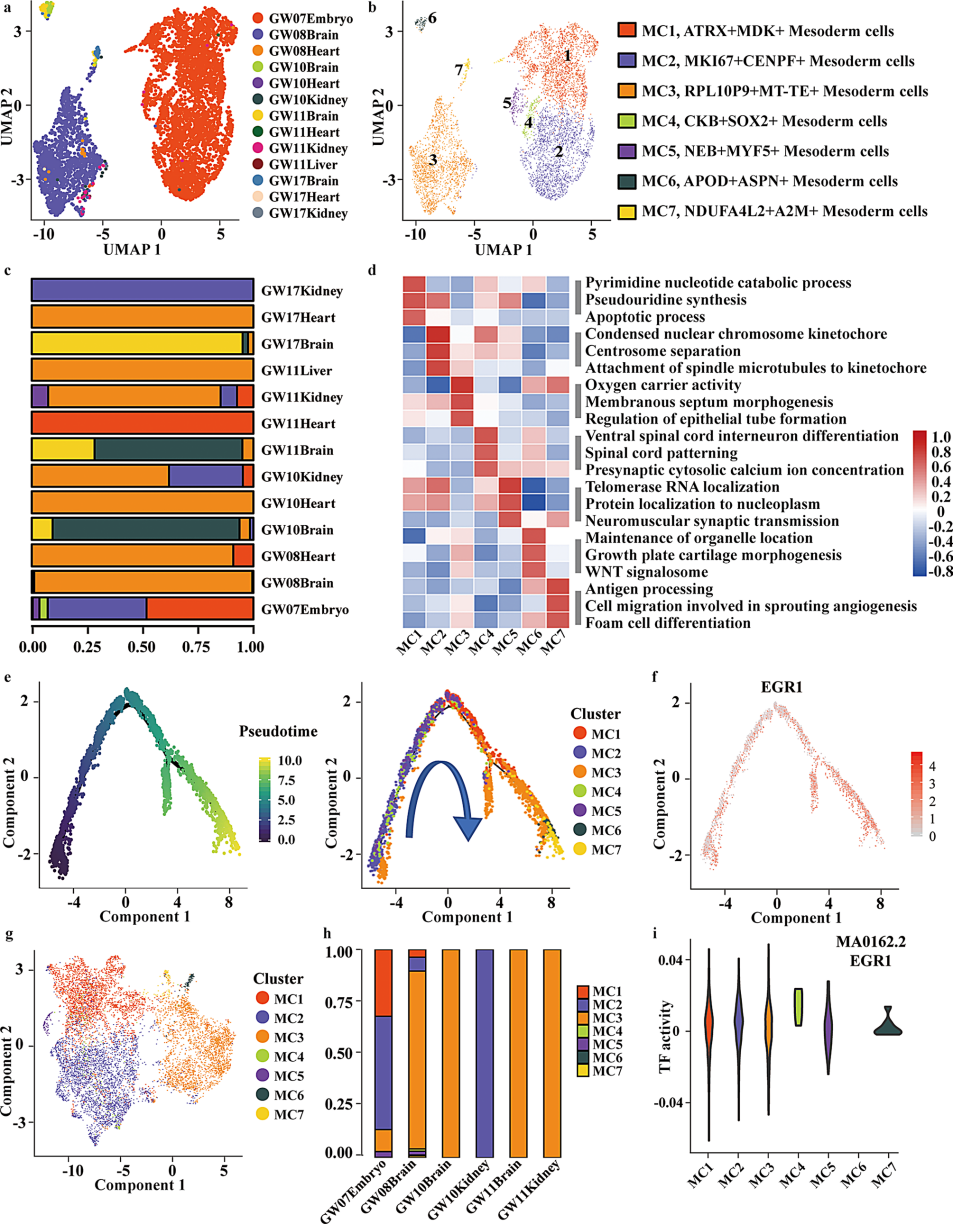
Heterogeneity and lineage inference of mesoderm cell (MC) sub-clusters. **a**, UMAP visualization of MCs derived from one GW07 embryo and five fetal organs (brain, heart, liver, kidney) across GW08–17, as analyzed by scRNA-seq. **b**, Reclustering of MCs identified seven distinct sub-clusters: MC1 (*ATRX*^+^*MDK*^+^), MC2 (*MKI67*^+^*CENPF*^+^), MC3 (*RPL10P9*^+^*MT-TE*^+^), MC4 (*CKB*^+^*SOX2*^+^), MC5 (*NEB*^+^*MYF5*^+^), MC6 (*APOD*^+^*ASPN*^+^), and MC7 (*NDUFA4L2*^+^*A2M*^+^). **c**, Proportions of MCs across developmental stages and tissues. **d**, Gene ontology (GO) enrichment analysis showing distinct biological processes associated with each mesodermal cluster (MC1-MC7). **e**, Trajectory analysis revealing a branched developmental trajectory of each mesodermal cluster (MC1-MC7). **f**, Expression pattern of representative transcription factor (TF) EGR1 along the trajectory. **g**, Single-cell open chromatin profiles of the seven MC sub-clusters. **h**, Cluster composition of MCs across different fetal organs. **i**, SCENIC analysis showing TF activity of EGR1 across MC1–MC7

To dissect the heterogeneity of MCs, we applied unsupervised clustering and pseudotime trajectory inference (Seurat v3.1.2 + Monocle2) across samples from GW07 to GW17 embryos (Table S5c). Cells were clustered based on the top 2,000 highly variable genes, and UMAP visualization revealed distinct transcriptional states. UMAP analysis revealed that MCs were most abundant in early embryonic samples (GW07) and progressively decreased in later-stage organ samples (GW08–GW17), suggesting that MCs are predominantly present in early development and subsequently differentiate into downstream organ-specific lineages (Fig. 2a).

Unsupervised clustering identified seven transcriptionally distinct MC sub-clusters (subset of MC cells) (MC1–MC7), each defined by specific marker genes, such as *ATRX* and *MDK* (MC1), *MKI67* and *CENPF* (MC2), *RPL10P9* and *MT-TE* (MC3), *CKB* and *SOX2* (MC4), *NEB* and *MYF5* (MC5), *APOD* and *ASPN* (MC6), and *NDUFA4L2* and *A2M* (MC7) (Fig. 2b, Fig. S3a). This data-driven approach ensured that cluster definition was based on statistically supported transcriptional signatures rather than arbitrary marker selection. Distribution analysis across developmental stages and tissues showed that MC1, MC2, MC4 and MC5 sub-clusters were enriched in early embryos (GW07), while MC3, MC6 and MC7 became predominant in later-stage organs (GW08-GW17) (Fig. 2c), supporting a temporal progression from proliferative progenitors to lineage-committed derivatives.

To investigate the biological processes and molecular functions of MC sub-groups, we analyzed the up-regulated genes of each MC sub-cluster versus other sub-clusters of MCs using GO enrichment. This method assesses whether specific GO terms occur more frequently in a given gene set than expected by chance, thereby highlighting functional pathways that characterize each sub-cluster. As a result, MC2 participated in centrosome separation and other cell-division functions. MC3 were active in the processes named membranous septum morphogenesis and epithelial tube formation, suggesting its possible role in the formation of the cardiovascular system. MC4 were associated with spinal cord patterning and presynaptic cytosolic calcium ion concentration regulation, suggesting it might be related to the formation of the spinal cord. Meanwhile, MC6 and MC7 were involved in WNT signalosome and sprouting angiogenesis, which suggested a relationship with the development of blood vessel cells (Fig. 2d). These enriched processes collectively suggest that MCs are hierarchically organized along organ-specific differentiation axes.

Trajectory analysis further revealed a continuous developmental progression inferred by Monocle2 pseudotime ordering (Fig. 2e). Early-stage MC2 and MC4 occupied the root of the trajectory, followed by MC5, MC1, and MC3, while later branches contained MC6 and MC7, which were transcriptionally primed toward lineage commitment and organ-specific differentiation. Monocle2 infers developmental trajectories by ordering single cells based on transcriptomic similarity using dimensionality reduction and minimum spanning tree (MST) construction, allowing reconstruction of continuous differentiation processes.

Subsequently, we sought to identify TFs potentially involved in MC differentiation. Using SCENIC analysis, we first obtained the top 10 enriched TFs in each MC sub-cluster (Fig. S3b) and overlapped them with the top 100 genes dynamically regulated along the inferred developmental trajectory. SCENIC reconstructs gene regulatory networks by integrating co-expression modules with motif enrichment analysis, thereby inferring active TFs and their downstream regulons at the single-cell level. Notably, only EGR1 overlapped between these two sets. Moreover, EGR1, together with 28 of its predicted target genes identified by SCENIC using the cisTarget database, was among the top 100 genes dynamically regulated along pseudotime (Fig. S3c), highlighting EGR1 as a central candidate regulator. The expression of EGR1 exhibited dynamic changes along pseudotime (Fig. 2f), supporting their involvement in MC fate transitions. To investigate the functional role of EGR1, we performed KEGG pathway enrichment analysis based on its 28 downstream target genes. These target genes were significantly enriched in pathways related to cell cycle regulation, p53 signaling, and oocyte meiosis (Fig. S3d), suggesting that EGR1 may coordinate cell proliferation and developmental signaling during mesodermal lineage commitment.

To further investigate transcriptional regulation, we performed scATAC-seq analysis of MCs (Table S5d). Integration of scRNA-seq and scATAC-seq further demonstrated highly concordant clustering, with mesodermal sub-clusters overlapping extensively in the joint UMAP space (Fig. S3e). Chromatin accessibility profiles confirmed the presence of seven MC sub-clusters (Fig. 2g), consistent with scRNA-seq clustering. Distribution analysis across tissues showed that early-stage embryos were enriched for MC1, MC2, MC3 and MC5 (GW07) (Fig. 2h). Importantly, TF motif enrichment revealed strong accessibility at the EGR1 motif across MC sub-clusters, except for MC6 (Fig. 2i). These findings provide orthogonal support from the chromatin landscape that EGR1 acts as a probable key regulator during MC differentiation and late-stage lineage specification.

### Lineage inference of MCs into cardiac cells

**Fig. 3:**
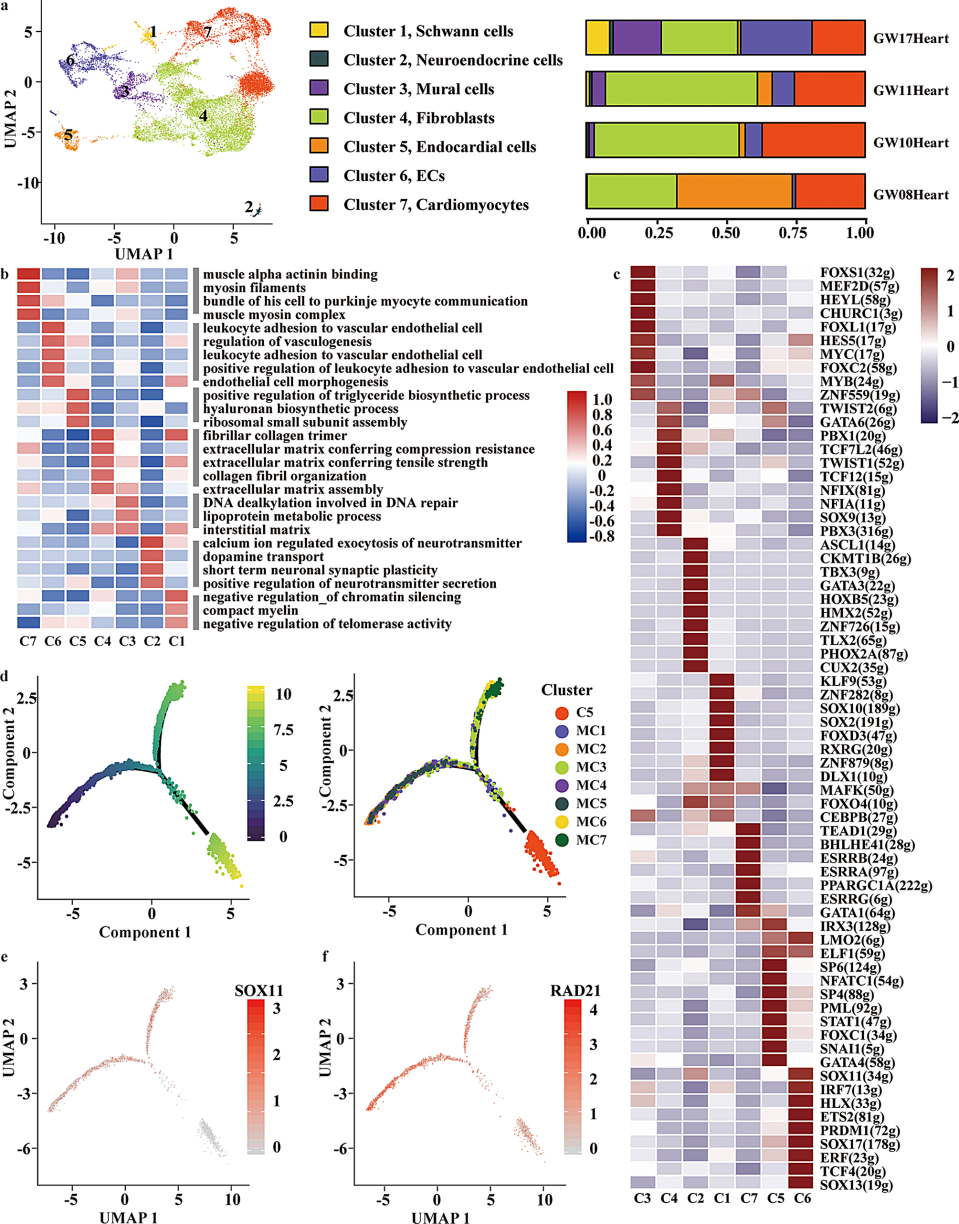
Lineage inference of MCs into cardiac cells. **a**, UMAP plot (left) showing the single-cell transcriptome landscape of cardiac-related cells (excluding hematopoietic cells) across four embryos (GW08, GW10, GW11, and GW17); each dot represents one cell. Clusters 1–7 correspond to Schwann cells (C1), Neuroendocrine cells (C2), Mural cells (C3), Fibroblasts (C4), Endocardial cells (C5), ECs (C6), Cardiomyocytes (C7). Right: bar plot showing the proportion of these clusters across different gestational stages. **b**, Gene Set Variation Analysis (GSVA) heatmap showing pathway activities of cardiac cell types. Values are expressed as row Z-scores of GSVA enrichment. **c**, Heatmap showing the top 10 TFs with the highest SCENIC activity scores (AUC values) in each cell type. Within each cell type, TFs are ranked in descending order of activity scores. Numbers previously shown in parentheses (e.g., 32 g) correspond to regulon IDs used by SCENIC/cisTarget for internal ranking. **d**, Monocle2-inferred trajectory of MCs and C5 (endocardial cells). Black solid lines represent the developmental backbone inferred by Monocle2. **e**-**f**, Violin plot showing the expression of *SOX11* and *RAD21* in the seven MC sub-clusters and C5, respectively. P-value was calculated with Wilcox likelihood-ratio test. *, *p* < 0.05; **, *p* < 0.01; ***, *p* < 0.001; ****, *p* < 0.0001

Mesodermal cardiogenesis originates from multiple cell lineages, including the first and second heart field (FHF and SHF), as well as multipotent neural crest cells [26, 27]. To further elucidate the regulatory mechanisms underlying this process, we next analyzed the transcriptional dynamics and lineage trajectories of MCs contributing to early cardiac development. Apart from hematopoietic cells, cardiomyocytes and fibroblasts are the main cell types in the heart. Consistently, we captured Schwann cells (C1), neuroendocrine cells (C2), mural cells (C3), fibroblasts (C4), endocardial cells (C5), endothelial cells (C6), and cardiomyocytes (C7) using the scRNA-Seq analysis (Fig. 3a). The identity of each cluster was validated by canonical marker expression (Fig. S4a).

To further characterize their functional properties, we performed GSVA enrichment analysis (Fig. 3b). Cardiomyocytes (C7) were strongly associated with muscle α-actinin binding, myosin filament, and muscle myosin complex, reflecting contractile function. Endothelial and endocardial clusters (C5–C6) were enriched in leukocyte adhesion, vasculogenesis, and endothelial morphogenesis, consistent with vascular development. Fibroblasts (C4) showed enrichment in extracellular matrix organization and collagen fibril assembly, highlighting their structural and matrix-producing roles. Neuroendocrine and Schwann cells (C1–C2) exhibited enrichment for synaptic signaling, neurotransmitter secretion, and neuronal plasticity, in line with their neural crest origin.

Besides, we identified cell-type-specific TFs using SCENIC analysis (Fig. 3c). For example, FOXC2, previously reported to regulate mural cell differentiation and vascular development [28], showed the highest activity in mural cells (C3) in our dataset. This finding not only validates our annotation strategy but also highlights FOXC2 as a conserved regulator of mural cell identity and function. Meanwhile, as proclaimed by other studies, TEAD1 promotes the differentiation of vascular smooth muscle cells and is necessary for mitochondrial functions in cardiomyocytes [29, 30]. In our results, we also discovered a special activity of TEAD1 in cardiomyocytes (C7).

To unveil which sub-cluster of MCs had the potential to develop into heart cells, we performed the trajectory analysis on all seven MC sub-clusters (MC1–MC7), together with endocardial cells (C5), ECs, and cardiomyocytes (Fig. 3d). Trajectory analysis revealed a branched developmental trajectory (Fig. 3d, left). When annotated by cluster identity, C5 (endocardial cells) localized at one trajectory branch, whereas different MC sub-clusters were distributed along intermediate states (Fig. 3d, right; Fig. S4b). Among them, MC3 was positioned adjacent to C5 (endocardial cells), suggesting a transcriptional proximity to endocardial lineage states.

Next, we explored the TFs potentially involved in regulating the transcriptional transition or lineage proximity between MC3 and C5 (endocardial cells). Based on SCENIC analysis, *SOX11* showed as the most active TF in MC3 (Fig. S4c). while the downstream genes of *SOX11*, including *EMCN* and *TMEM100*, were highly expressed in C5 (endocardial cells) compared to MC1-7 sub-groups (Fig. S4d-e). Meanwhile, we found that *SOX11* was predominantly expressed in MCs and decreased in C5 cells compared to MCs (Fig. 3e). Given previous evidence implicating *SOX11* in cardiac differentiation [31], these results point to a potential role of *SOX11* in linking MC progenitor states (such as MC3) with endocardial cell programs.

On the other hand, SCENIC analysis revealed that five putative RAD21 target genes (*EMCN*,* POSTN*,* ECSCR*,* HBB*, and *RNASE1*) were highly expressed in C5 (endocardial cells) but not in mesodermal clusters (MC1–7) (Fig. S4e). Several of these genes, including *POSTN* and *EMCN*, are well-established regulators of cardiac development and endothelial function [32, 33], suggesting that this regulatory module may specifically define endocardial identity. Along the developmental trajectory, *RAD21* expression was detected not only in MCs but also in C5 endothelial cells (Fig. 3e), implying a role that spans from mesodermal progenitors to endocardial differentiation. Consistently, previous studies have shown that *RAD21* depletion causes cardiac defects in zebrafish [34], underscoring its conserved role in heart development. Taken together, these findings suggested that *SOX11* and *RAD21* may serve as key transcriptional regulators in the specification and maintenance of endocardial cells.

### Lineage inference of MCs into ECs

**Fig. 4:**
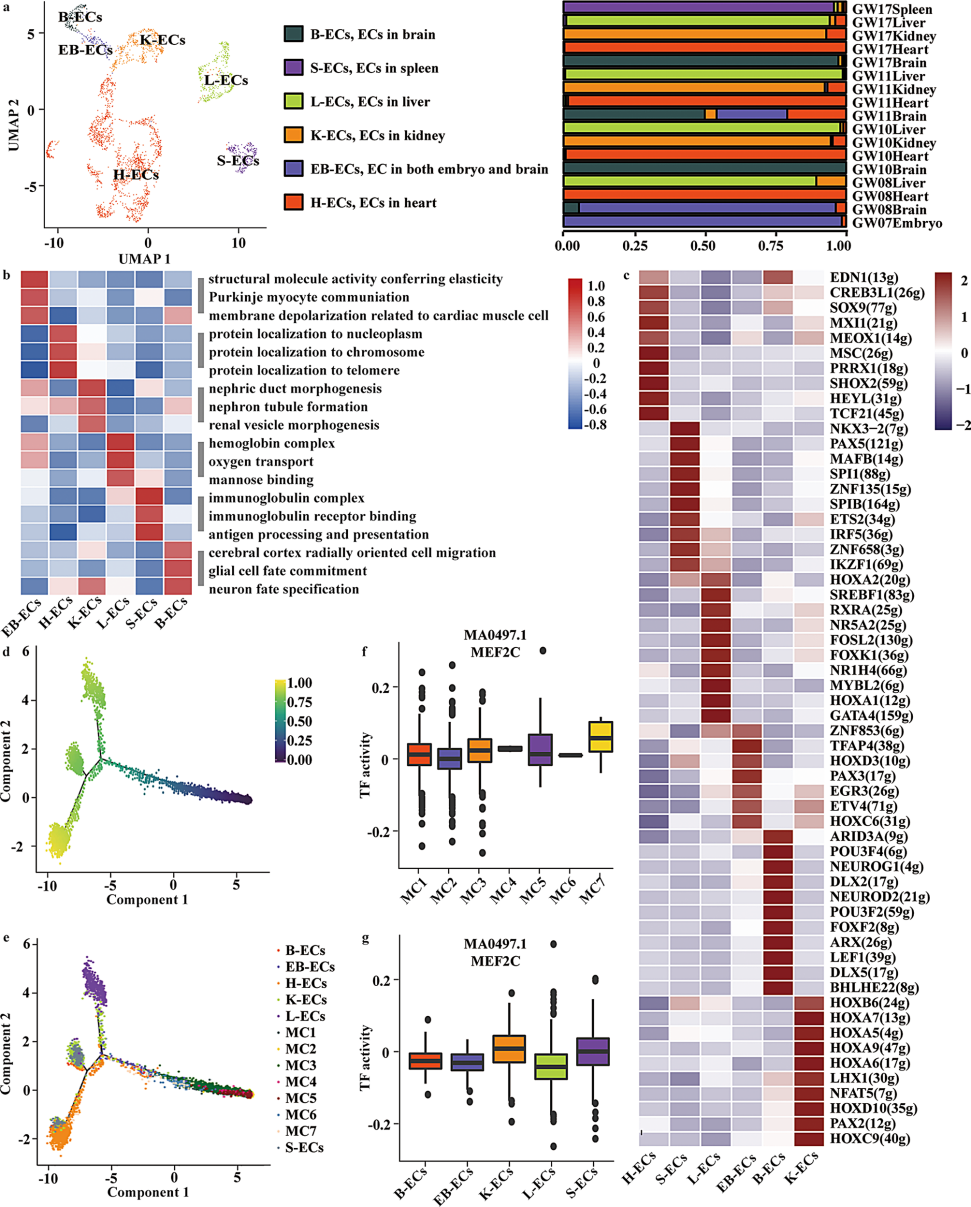
Lineage inference of MCs into endothelial cells (ECs). **a**, UMAP plot showing six EC clusters from multiple tissues and stages (each dot = one cell). Clusters include: B-ECs (brain ECs), H-ECs (heart ECs), K-ECs (kidney ECs), L-ECs (liver ECs), S-ECs (spleen ECs), and EB-ECs (embryo–brain ECs). Right: bar plot showing relative abundance of EC clusters per sample/gestational stage. **b**, Heatmap showing functional pathway enrichment among six EC sub-clusters (H-ECs, K-ECs, L-ECs, S-ECs, B-ECs, EB-ECs), scored by GSVA. **c**, Heatmap showing the top 10 active TFs with the highest SCENIC activity scores (AUC values) in each EC cluster. TFs are ranked within each cluster by activity. Numbers previously shown in parentheses (e.g., 32 g) correspond to regulon IDs used by SCENIC/cisTarget for internal ranking. **d**, developmental trajectories were reconstructed using Monocle2 with the DDRTree algorithm. Pseudotime ordering revealed a continuous developmental progression with three major branches, representing transitions from early MCs to differentiated endothelial states. The color scale indicates pseudotime progression from early (blue) to late (yellow) stages. **e**, The same trajectory colored by cell identity shows that early pseudotime states are dominated by MC2 and MC4, whereas distal branches contain endothelial subtypes (B-ECs, EB-ECs, H-ECs, K-ECs, L-ECs, and S-ECs). **f**-**g**, Boxplots showing TF activity of MEF2C in MC and EC sub-clusters (MC1–MC7). Statistics: two-sided Wilcoxon rank-sum test with multiple-testing correction

The mesoderm possesses the remarkable ability to differentiate into ECs, which constitute the inner lining of blood vessels, playing a crucial role in the formation of the vascular network and active participation in blood circulation [35]. To explore the sub-cluster(s) of MCs developing into ECs, we next focused on ECs to characterize their identity and heterogeneity. scRNA-seq analysis with UMAP embedding revealed ECs distributed across multiple embryonic organs and developmental stages (Fig. S5a). The expression of canonical and subtype-associated markers, including *FABP4*,* IGFBP5*,* AL513542.1*,* CDH5*,* OIT3*, and *MFSD2A* further confirmed endothelial identity and highlighted transcriptional diversity within the population (Fig. S5b). To resolve organ-specific heterogeneity, ECs were subsequently classified into six clusters: heart ECs (H-ECs; *FABP4*^+^), kidney ECs (K-ECs; *IGFBP5*^+^*EHD3*^+^), liver ECs (L-ECs; *OIT3*^+^), spleen ECs (S-ECs; *AL513542.1*^+^*NKX2-3*^+^), brain-specific ECs (B-ECs; *MFSD2A*^+^), and embryo–brain ECs (EB-ECs; *CDH5*^+^) (detected both in the brain tissue and in the whole-embryo at GW07), which were consistent with the previous study [3] (Fig. 4a, Table S5e).

Functional enrichment analysis using GSVA implied that these EC subtypes were associated with biological processes relevant to their tissue origins, such as vascular morphogenesis, oxygen transport, immune binding, and renal vesicle morphogenesis (Fig. 4b). In detail, kidney ECs (K-ECs, *IGFBP5 + EHD3 +* ECs) were active in the nephric duct and renal vesicle development, liver ECs (L-ECs, *OIT3 +* ECs) were involved in mannose-binding, spleen ECs (S-ECs, *AL513542.1 + NKX2-3 +* ECs) were associated with immunoglobulin receptor binding. Brain ECs had two sub-clusters of cells, one cluster of ECs only located in the brain (B-ECs, *MFSD2A +* ECs), and the other cluster of ECs was identified in both brain and the entire 7-week-age embryo (EB-ECs, *CDH5 +* ECs). B-ECs were active in neuron fate specification.

SCENIC analysis revealed distinct TF activity patterns across EC sub-clusters (Fig. 4c). H-ECs showed strong activation of SOX9, consistent with cardiac and vascular development [36]. S-ECs were enriched for PAX5, key regulator of B-cell differentiation and splenic white pulp development [37]. L-ECs exhibited activity of GATA4, in line with its requirement for liver endothelial differentiation and embryonic hematopoiesis [38]. B-ECs exhibited activation of LEF1, indicative of Wnt signaling–mediated regulation of brain vasculature development and blood–brain barrier (BBB) properties [39]. While K-ECs were marked by PAX2, a well-established TF guiding nephron progenitor specification and kidney development [40]. Together, these TF profiles delineate organ-specific regulatory networks underlying endothelial heterogeneity. Together, these results uncover candidate TFs that may govern the differentiation of MCs into organ-specific endothelial subtypes, thereby highlighting key regulatory programs underlying endothelial heterogeneity.

To clarify the potential origins of EC sub-clusters from MCs, trajectory analysis was performed to reveal the developmental continuum between mesodermal and endothelial lineage**s(**Fig. 4d-e, Fig. S5c). Using Monocle2 with the DDRTree algorithm, we reconstructed single-cell developmental trajectories based on transcriptional similarity. The inferred pseudotime ordering revealed three major branching paths (Fig. 4d), representing progressive transcriptional transitions from early MCs to differentiated endothelial subtypes. When colored by cell identity (Fig. 4e), early pseudotime states were predominantly composed of mesodermal sub-clusters (MC1 - MC7), particularly MC2 and MC4, which occupied the root of the trajectory. In contrast, distal branches were enriched for EC subtypes, including B-ECs, EB-ECs, H-ECs, K-ECs, L-ECs, and S-ECs, corresponding to more advanced differentiation states. Notably, MC7 was positioned adjacent to EB-ECs, indicating the strongest transcriptional continuity between these two populations. This spatial proximity along the trajectory suggests that MC7 may represent a mesodermal population transcriptionally primed toward endothelial or hemogenic fate, thereby bridging the transition from mesodermal to vascular lineages during human embryogenesis.

Next, to identify key regulators potentially involved in the differentiation of MC7 toward endothelial lineages, we examined transcriptional profiles and found that *MEF2C* expression was significantly higher in MC7 than in kidney and spleen endothelial subtypes (K-ECs and S-ECs), and comparable to brain ECs (B-ECs) (Fig. S5d). Furthermore, SCENIC analysis identified *ANGPT2* and *COL4A2* as putative MEF2C-target genes. A subsequent GO enrichment analysis of these MEF2C-targeted genes revealed significant enrichment in terms related to angiogenesis and vascular system development. (Fig. S5e), consistent with previous studies implying the essential role of MEF2C in vascular system development and angiogenesis [41]. These findings are in line with previous reports highlighting the indispensable role of MEF2C in vascular formation and angiogenesis [41]. Moreover, SCENIC analysis revealed that MEF2C was dynamically regulated across both MCs and ECs (Fig. 4f–g). Among MCs, MEF2C activity was most prominent in MC7 (Fig. 4f), consistent with its potential role in late-stage mesodermal differentiation. Across EC sub-clusters, elevated MEF2C activity was observed particularly in EB-ECs and K-ECs, suggesting its involvement in endothelial lineage specification and organ-specific vascular development (Fig. 4g). Together, these findings imply MEF2C as a potential key regulator bridging MCs with EC differentiation.

### Hematopoietic differentiation trajectories from mesodermal progenitors

**Fig. 5:**
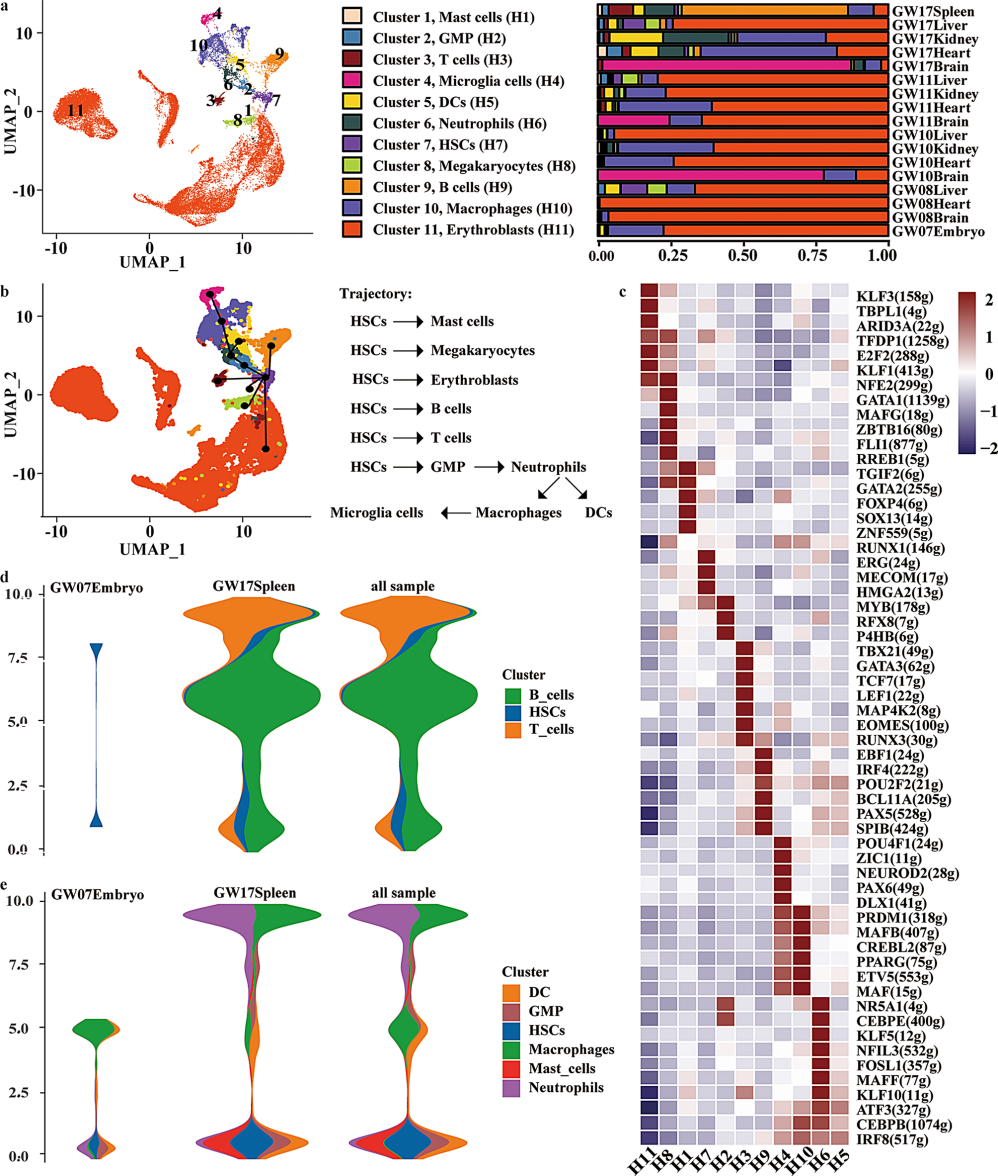
Lineage inference of MCs into hematopoietic stem cells (HSCs). **a**, UMAP plot (left) of hematopoietic cells captured from fetal heart, kidney, liver, brain, spleen, and a 7-week embryo across gestational weeks 7–17. Each dot represents one single cell, colored by cluster identity. Eleven major clusters were identified: mast cells (H1), granulocyte–macrophage progenitors (GMP, H2), T cells (H3), microglia cells (H4), dendritic cells (DCs, H5), neutrophils (H6), hematopoietic stem cells (HSCs, H7), megakaryocytes (H8), B cells (H9), macrophages (H10), and erythroblasts (H11). Bar plot (right) showing the relative proportions of each hematopoietic cluster across different tissues and developmental stages (GW07–17). **b**, Monocle2 trajectory analysis of hematopoietic cells, showing developmental progression from HSCs toward differentiated lineages. Black solid lines indicate the inferred developmental backbone. **c**, Heatmap showing the top 10 TFs with the highest SCENIC activity scores (AUC values) in each hematopoietic cell type. TFs are ranked by activity within each cell type. Parentheses with numbers in the original version (e.g., “32 g”) indicated regulon IDs used internally by SCENIC. **d**-**e**, Pseudotime trajectory of HSCs differentiating toward lymphoid lineages in GW07 embroy, GW17 spleen and pooled samples

HSCs originating from MCs have been well-characterized, which differentiate into erythroid cells, lymphoid cells, and myeloid cells [42]. To figure out the unique sub-cluster(s) of MCs into HSCs, we first clustered hematopoietic cells from the heart, kidney, liver, brain, spleen. As expected, we identified 11 distinct clusters (H1–H11). These included mast cells (H1), granulocyte–monocyte progenitors (GMPs, H2), T cells (H3), microglial cells (H4), dendritic cells (DCs, H5), neutrophils (H6), hematopoietic stem cells (HSCs, H7), megakaryocytes (H8), B cells (H9), macrophages (H10), and erythroblasts (H11) (Fig. 5a).

CytoTRACE analysis was applied to assess developmental potential across hematopoietic clusters. HSCs (H7) and megakaryocytes (H8) exhibited the highest entropy values (Fig. S6a), consistent with their primitive and multipotent states. In contrast, terminally differentiated lineages such as neutrophils (H6) and macrophages (H10) showed the lowest entropy, reflecting restricted developmental plasticity (Fig. S6a). Trajectory inference using Slingshot projected potential developmental paths onto the UMAP embedding (Fig. 5b), revealing putative lineage relationships among the identified cell clusters. The identified clusters of cells showed a consistent differentiation trajectory.

We observed the highest expression of *HBZ* in erythroblasts along the pseudo-time trajectory (Fig. S6b). As a control, we simultaneously detected the expression of other HB family genes, and found *HBQ1*, *HBM*, and *HBG1* were expressed in the blood system cells (Fig. S6c). The previous report showed that *HBZ* was transcribed in primitive erythroid cells in embryonic development [43]. Consistently, our data confirmed enriched *HBZ* expression in embryonic erythroblasts, underscoring its role as a marker of primitive erythropoiesis.

Using SCENIC analysis, we verified TFs specifically active in each type of hematopoietic cells (Fig. 5c). Consequently, the SCENIC results were consistent with the previous reports. For example, we found E2F2 was most active in erythroid cells. Many studies have demonstrated that E2F2 was functional in the terminal maturation of erythroid cells [44]. TBX21 and EOMES exhibited specific activity in T cells, consistent with their established roles as canonical regulators of T cell lineage commitment and effector function [45]. Similarly, MAFB showed predominant activity in macrophages, in line with previous reports that MAFB is essential for macrophage differentiation and functional maturation [46]. The concordance between our findings and prior studies highlights the reliability of our SCENIC-based TF inference and underscores the conserved roles of these lineage-defining TFs in the establishment of immune cell identity during human embryonic development.

To specifically investigate hematopoietic lineage commitment in the fetal spleen, we reconstructed the differentiation trajectories of HSCs (Fig. 5d–e). Analysis revealed two major branches. The first branch led to lymphoid fates, with HSCs giving rise to B cells and T cells, which became prominent in the fetal spleen by GW17 (Fig. 5d). The second branch followed a myeloid program, in which HSCs differentiated into dendritic cells, GMPs, macrophages, mast cells, and neutrophils (Fig. 5e). These findings delineate the bifurcated developmental potential of HSCs, establishing distinct lymphoid and myeloid lineages during fetal hematopoiesis.

## Discussion

This study systematically delineates the developmental landscape of MCs from gestational weeks 7 to 17 using large-scale single-cell transcriptomic and chromatin accessibility profiling. Our analysis reveals the differentiation trajectories of MCs during the organogenesis of multiple key tissues and identifies a set of genes that may play critical regulatory roles in lineage specification and organ development. In contrast to previous studies that were primarily based on animal models or mid-gestational human embryos [3], our work provides, for the first time, a comprehensive single-cell–level insight into mesodermal development in early to mid-gestational human embryos through the integration of transcriptomic and chromatin accessibility data.

To achieve this, we combined scRNA-seq and scATAC-seq to explore the transcriptional programs and regulatory networks underlying mesodermal differentiation. While these two technologies provide complementary information, they also differ in data resolution and biological interpretation. scRNA-seq captures transcriptional outputs at a given time point, whereas scATAC-seq provides insights into chromatin accessibility that reflects potential regulatory activity. Thus, direct comparison should be made with caution, as transcript abundance does not always correspond to chromatin openness. Nevertheless, the integration of these two datasets enabled us to cross-validate key findings. For example, TFs such as EGR1 not only showed transcriptional enrichment in scRNA-seq but also exhibited motif accessibility in scATAC-seq, strengthening the robustness of our conclusions. Taken together, although technical and biological biases inherent to each method exist, their combined use offers a synergistic view of cell fate regulation and increases confidence in the identified regulatory relationships.

Consistent with their transcriptional features, GO enrichment analysis further substantiated the functional diversity of the MC1–7 subclusters. MC2, characterized by *MKI67* and *CENPF*, was enriched for terms such as centrosome separation, chromosome segregation, and mitotic cell cycle, reflecting its proliferative and expansion-associated state rather than a distinct lineage identity. MC3 was enriched for cardiac muscle tissue development and sarcomere organization, supporting its cardiogenic priming; MC4 showed enrichment for neural tube morphogenesis and axial patterning, indicating a neuro-mesodermal tendency; MC5 for skeletal muscle differentiation; MC6 for extracellular matrix organization and angiogenesis; and MC7 for blood vessel morphogenesis and hemopoiesis-related processes, consistent with its endothelial/hemogenic priming and MEF2C activity. Together, these results suggest that MC1–7 represent a developmental continuum of mesodermal progenitors transitioning from proliferative to lineage-biased states, with GO analysis providing statistical support for their distinct biological roles, while functional validation will be needed to confirm these inferred identities.

By integrating trajectory and CytoTRACE analyses, we inferred a putative progression order among mesodermal sub-clusters (MC2 > MC4 > MC5 > MC1 > MC3 > MC6 > MC7) (Fig. S6d). Our results suggest that MC2 represents a proliferative expansion pool rather than the earliest mesodermal founder, while MC4 and MC5 reflect neuro-axial and myogenic specification. MC1 appears as a transitional regulatory state, MC3 shows cardiovascular priming, and MC6 carries angiogenic–stromal features. Importantly, MC7 exhibits strong MEF2C activity and is positioned close to EC-related trajectories, suggesting potential involvement in endothelial programs. True primitive progenitors may lie upstream of MC2 but were not captured in our GW07–17 samples. Despite limitations from sampling windows and modality resolution, concordant TF activity (e.g., MEF2C, EGR1) provides supportive evidence for these interpretations.

In this study we concentrated on mesodermal trajectories leading to heart cells, ECs, and HSCs, as these lineages were most clearly resolved from our single-cell datasets. Nevertheless, other mesoderm-derived tissues such as skeletal muscle, bone, kidney, and gonads are also of great developmental interest. For instance, MC5 (*NEB*^+^*MYF5*^+^) showed myogenic signatures consistent with skeletal muscle progenitors [47], and MC4 exhibited neuro-mesodermal features potentially related to axial structures. However, our samples (GW07–17) did not provide sufficient resolution to reconstruct full trajectories toward bone, kidney, or gonadal lineages. This limitation likely reflects not only the developmental window of sample collection but also the restricted set of tissues profiled, the possibility that some rare progenitor states were under-sampled, and the inherent sparsity of single-cell multi-omics data, which may obscure low-abundance differentiation paths. Future studies incorporating earlier developmental stages, a broader range of tissues, and complementary approaches such as spatial transcriptomics and lineage tracing will be required to fully delineate these additional trajectories.

The identification of EB-ECs is particularly intriguing, as they were distributed both in the brain and throughout the GW07 embryo. Their dual presence raises the question of whether they constitute a homogeneous population or represent a mixture of distinct endothelial subsets. One possible explanation is that the EB-ECs captured from the entire GW07 embryo may have arisen from brain-derived endothelial progenitors at the same developmental stage, reflecting early dissemination or sampling overlap. Alternatively, EB-ECs may represent a transient endothelial subtype with broader developmental potential that is gradually regionalized during organogenesis. Further spatial and temporal analyses will be necessary to clarify whether EB-ECs are a unique homogeneous cell type or a heterogeneous mixture with tissue-specific origins.

Our single-cell analysis of human embryos (GW07–17) revealed that MCs exhibit marked heterogeneity and lineage biases toward cardiac, endothelial, and hematopoietic fates. These findings are broadly consistent with studies in mouse embryogenesis, which have established that MCs play central roles in cardiogenesis, vasculogenesis, and hematopoietic initiation, with distinct proliferative pools and lineage-primed sub-clusters [26, 35, 41]. Nevertheless, we also observed important human–mouse differences. In humans, the transition of MC sub-clusters toward endocardial and hematopoietic fates appeared more gradual, whereas mouse studies often describe more rapid and synchronous transitions at comparable stages. Moreover, human embryos showed the presence of sub-clusters such as MC3 with cardiovascular priming, while mouse mesoderm is typically classified into the first and second heart fields with clearer spatial demarcation [27]. Together, these results highlight conserved principles of mesodermal development across species while also emphasizing human-specific differences in timing, heterogeneity, and transitional states, underscoring the value of direct human embryonic datasets.

Our integrative analysis identified EGR1 as a central transcriptional regulator of MC differentiation. Previous studies have demonstrated its essential role in maintaining HSC quiescence and homing [48], as well as in orchestrating angiogenic signaling through the induction of VEGF and other vascular regulators [49]. Beyond the hematopoietic system, EGR1 has also been implicated in tissue remodeling, cardiac repair, and neural differentiation, reflecting its broad influence on lineage specification and regenerative responses [50]. Moreover, as a master regulator under ischemic and developmental stress, EGR1 coordinates multiple gene networks to balance proliferation with differentiation [51]. These findings suggest that EGR1 functions as a molecular hub at the intersection of proliferation, stress response, and differentiation programs. These findings suggest that EGR1 may play a regulatory role in the differentiation of MCs into distinct organ-specific lineages.

This study is not only of fundamental significance in developmental biology but also provides valuable insights for regenerative medicine and disease modeling. First, the developmental trajectories of human MCs and the associated transcriptional regulatory networks delineated here offer a theoretical framework for directed differentiation of stem cells and organoid construction in vitro. For instance, the trajectories from mesodermal sub-clusters toward cardiac, renal, and endothelial lineages may serve as references for optimizing protocols of induced pluripotent stem cell differentiation, thereby advancing applications in myocardial repair, kidney regeneration, and vascular engineering. Second, aberrant mesodermal differentiation is closely associated with a variety of congenital disorders, such as congenital heart disease, renal dysgenesis, and hematopoietic abnormalities. Our single-cell atlas not only highlights potential pathogenic mechanisms underlying these conditions but also provides cell-level targets for disease modeling and drug screening. Finally, with the rapid development of single-cell sequencing, spatial transcriptomics, and other multi-omics technologies, future efforts will likely integrate larger-scale human embryonic datasets across developmental stages. By incorporating epigenomic, spatial, and functional validation data, it will be possible to construct a more comprehensive and dynamic map of human development. Such advances will not only deepen our understanding of the mechanisms of human organogenesis but also provide a valuable resource for precision medicine and regenerative medicine research.

This study also has certain limitations. First, due to the difficulty of obtaining human embryonic samples, each developmental stage and organ typically included only one sample, and some rare cell populations were represented by a limited number of cells, which may affect statistical robustness. Second, single-cell transcriptomic and ATAC-seq data from early human embryonic tissues are characterized by relatively low numbers of detected genes or accessible chromatin regions per cell, which largely reflects the limited RNA content and intrinsic properties of such samples rather than technical shortcomings. Although this may restrict resolution in certain analyses, we applied stringent quality control, down-sampling validation, and multi-omics integration to minimize potential biases. Future studies with larger sample sizes, higher sequencing coverage, and more comprehensive datasets will be needed to strengthen the conclusions. Finally, this work is primarily descriptive in nature, and some of the inferred regulatory factors and differentiation relationships will require further validation through functional experiments in vitro and in vivo.

## Conclusion

In this study, we collected hearts, livers, spleens, kidneys, and brains of human embryos ranging from 7 to 17 gestational weeks of age (17 samples in total). We performed scRNA-Seq and ATAC-Seq to analyze the developmental features of MCs to mesoderm-derived cells. As a result, we discovered seven sub-clusters of MCs and uncovered the biological function differences between them. We then revealed the potential differentiation order of the seven clusters and identified EGR1 as a probably important TF for the differentiation. Subsequently, we characterized the differentiation of sub-clusters of MCs into downstream progenitor cells and provided a TF regulatory blueprint of mesoderm differentiation. Additionally, we revealed the differentiation trajectory of the above progenitor cells and proclaimed the potentially functional genes involved in the differentiation. Our study’s results will advance our knowledge of the early stages of embryonic development and open up fresh possibilities for organ regeneration bioengineering.

## Supplementary Information

Below is the link to the electronic supplementary material.

Supplementary Material 1

Supplementary Material 2

Supplementary Material 3

## Data Availability

The scRNA-seq datasets generated during this study are available at OMIX under accession number OMIX004136: https://ngdc.cncb.ac.cn/omix/release/OMIX004136. The scATAC-seq datasets generated during this study are available at OMIX under accession number OMIX004137: https://ngdc.cncb.ac.cn/omix/release/OMIX004137. Any additional information required to reanalyze the data reported in this paper is available from the lead contact upon request.
