# Supplementary material for: Molecular architecture of mesoderm cells across early to middle stage of human embryo development at single-cell resolution
Source: BMC Mol Cell Biol. 2025 Dec 25;27:3. doi: 10.1186/s12860-025-00561-9 (PMC12849250; doi:10.1186/s12860-025-00561-9)
Supplement: Supplementary file 1 — Supplementary Material 1 [file 12860_2025_561_MOESM1_ESM.docx]

**Supplementary Fig. S1**


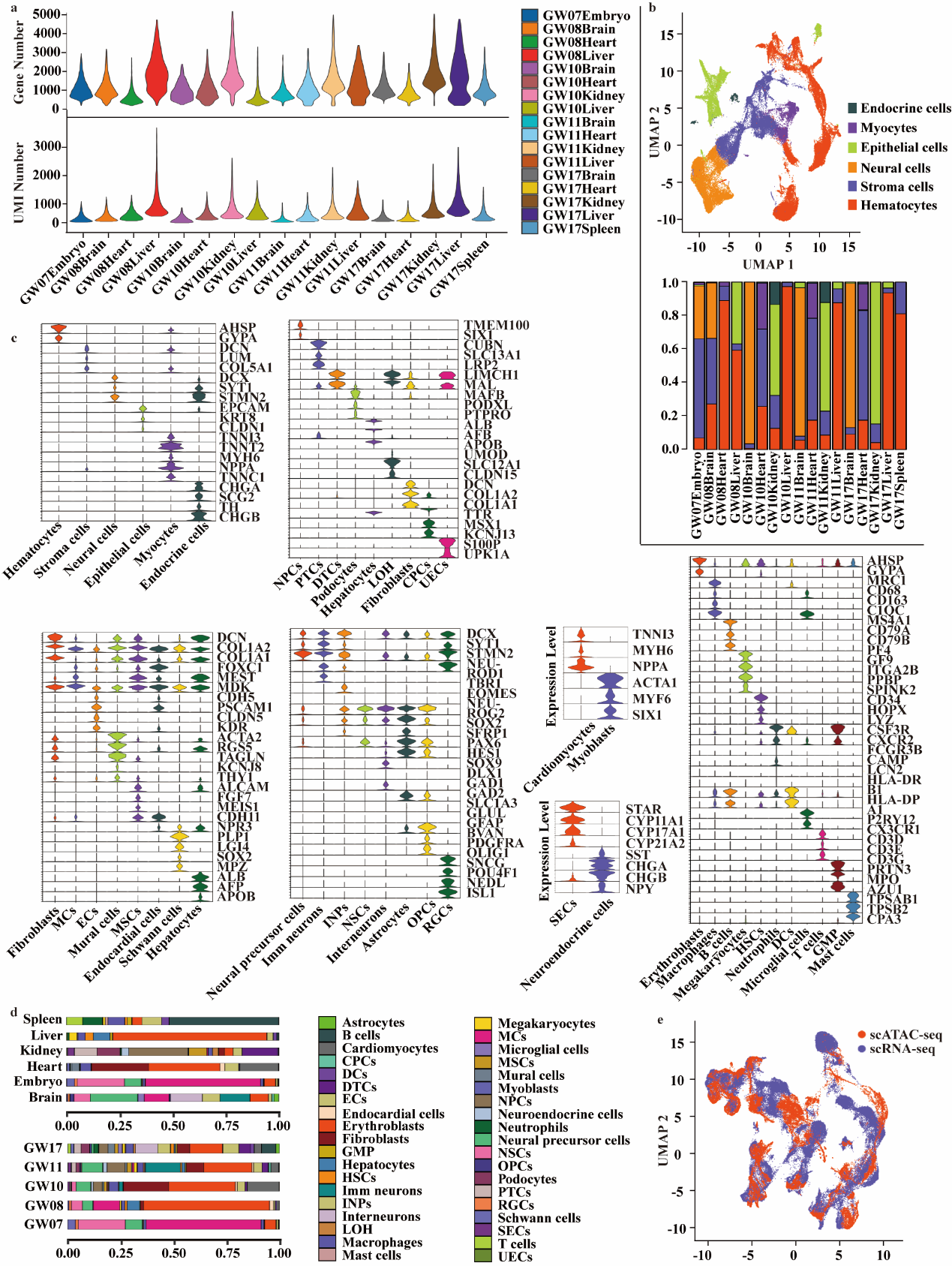


**Supplementary Fig. S2**


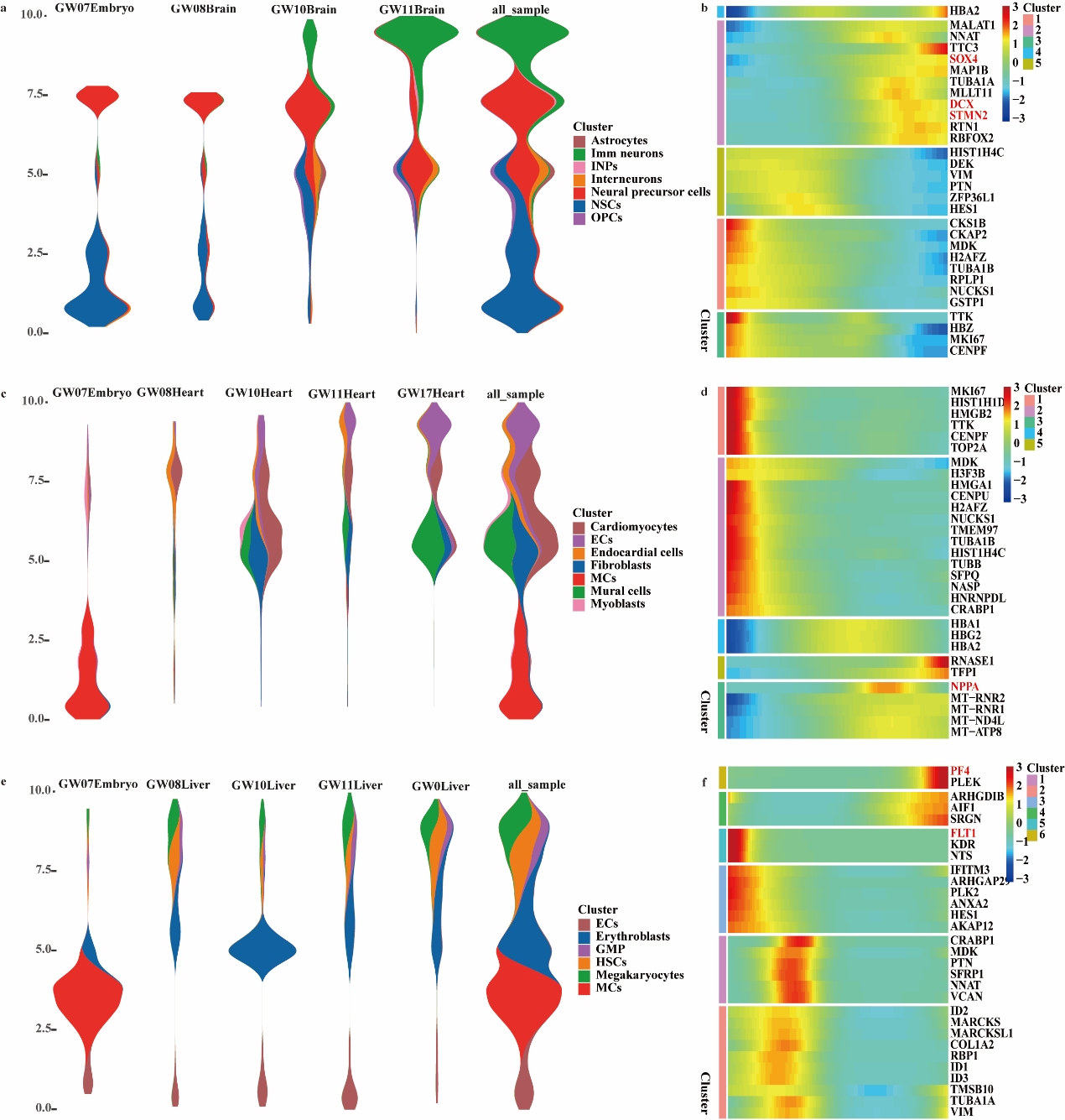


**Supplementary Fig. S3**


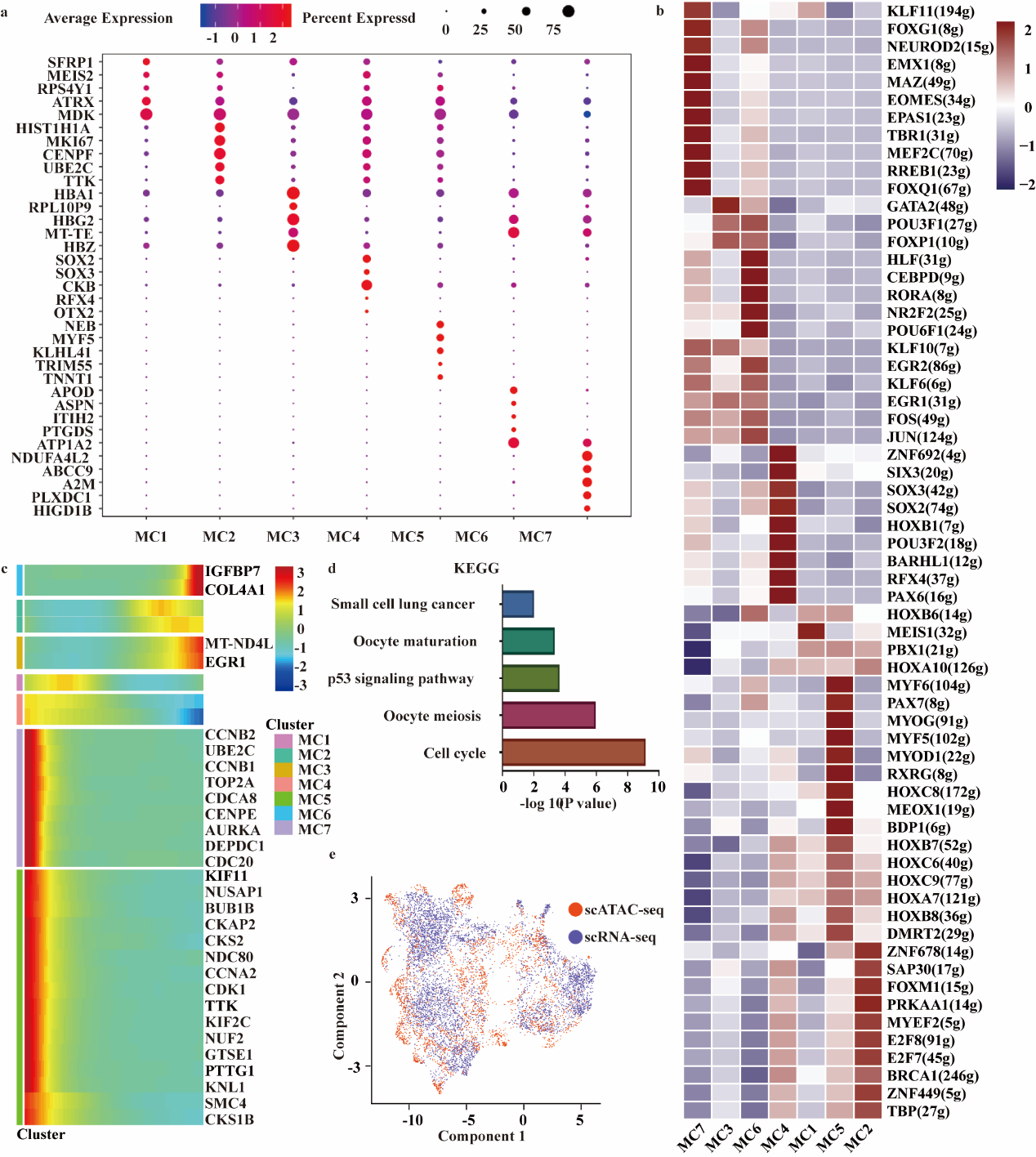


**Supplementary Fig. S4**


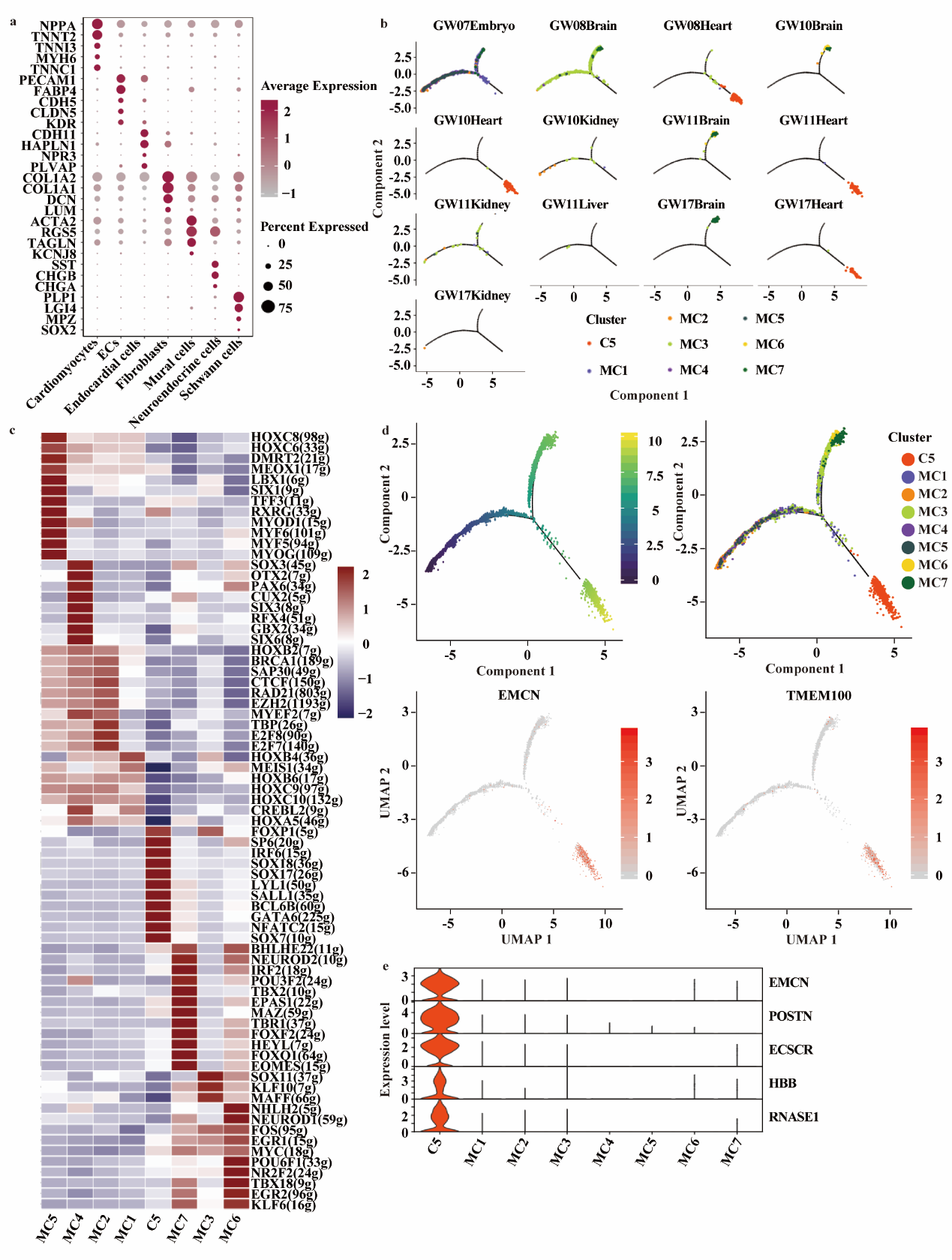


**Supplementary** **Fig. S5**


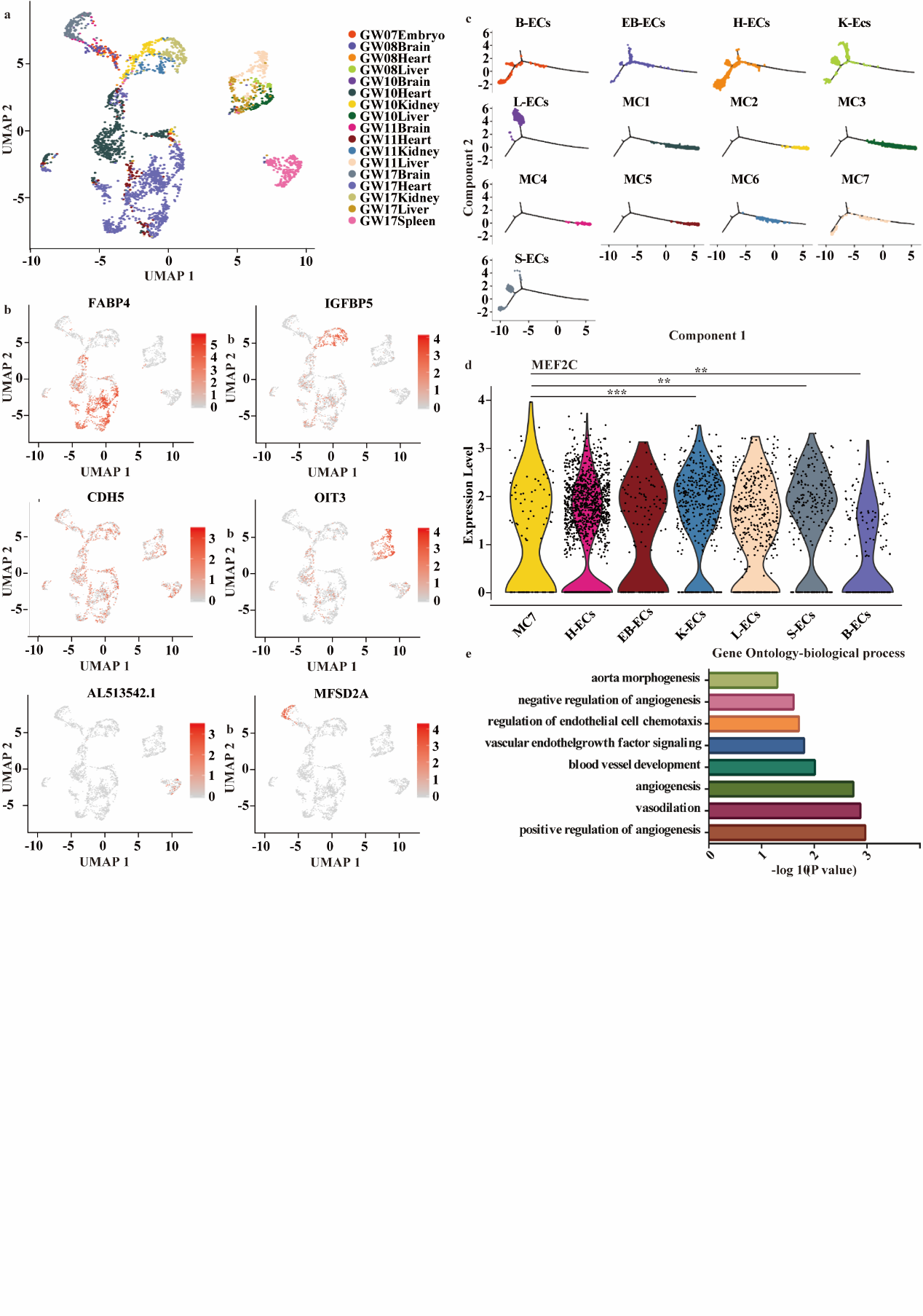


**Supplementary Fig. S6**


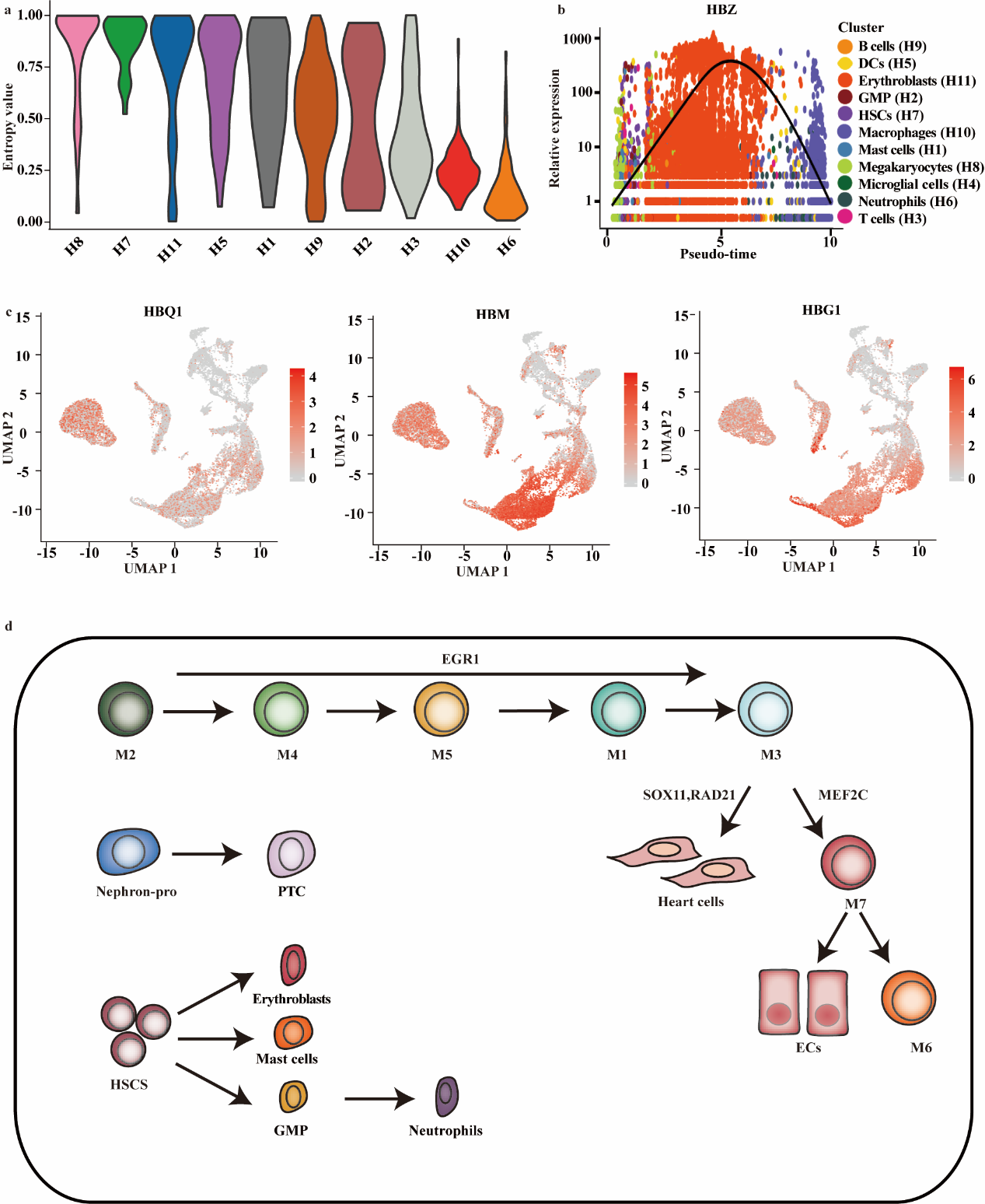


**Supplementary Table S1. Summary of software, databases, and tools**

| **Method / Software / Database** | **Version** | **Function / Purpose** | **Official URL** |
| --- | --- | --- | --- |
| Cell Ranger | v3.0.x / v3.1.0 | Generate single-cell gene expression matrices | <https://www.10xgenomics.com> |
| Cell Ranger ATAC | v1.2.0 | Processing scATAC-seq (single-cell ATAC sequencing) data | <https://support.10xgenomics.com/single-cell-atac/software/overview/welcome> |
| STAR aligner | v2.7.3a | RNA-seq read alignment | <https://github.com/alexdobin/STAR> |
| Seurat | v3.1.2 | Preprocessing, clustering, and dimensionality reduction | <https://satijalab.org/seurat/> |
| MACS2 | v2.1.1 | Peak calling for ATAC-seq data | <https://github.com/macs3-project/MACS> |
| SnapATAC | v1.0.0 | Single-cell ATAC-seq analysis | <https://github.com/r3fang/SnapATAC> |
| HOMER | v4.11 | Motif enrichment analysis | <http://homer.ucsd.edu/homer/> |
| Monocle2 | v2.x | Pseudotime trajectory inference | <http://cole-trapnell-lab.github.io/monocle-release/> |
| Slingshot | v1.4.0 | Trajectory reconstruction using MST | <https://bioconductor.org/packages/slingshot/> |
| SCENIC | v1.1.2-3 | TF regulatory network inference | <https://github.com/aertslab/SCENIC> |
| GENIE3 | v1.6.0 | Co-expression based regulatory network inference | <https://bioconductor.org/packages/GENIE3/> |
| RcisTarget | v1.6.0 | Motif enrichment and target prediction | <https://bioconductor.org/packages/RcisTarget/> |
| AUCell | v1.6.0 | Regulon activity quantification | <https://bioconductor.org/packages/AUCell/> |
| chromVAR | v1.8.0 | TF motif activity analysis for ATAC-seq | <https://bioconductor.org/packages/chromVAR/> |
| AnimalTFDB | — | Transcription factor annotation database | <http://bioinfo.life.hust.edu.cn/AnimalTFDB4/> |
| CytoTRACE | v0.3.3 | Estimate cell differentiation potential | <https://cytotrace.stanford.edu> |
| SLICE | v0.99.0 | Calculate transcriptional entropy for stemness | <https://bioconductor.org/packages/SLICE/> |
| clusterProfiler | v3.16.1 | GO/KEGG pathway enrichment | <https://bioconductor.org/packages/clusterProfiler/> |
| GSVA | v1.36.3 | Estimate pathway activity variation | <https://bioconductor.org/packages/GSVA/> |
| Jaccard similarity | — | Quantify overlap between gene sets | — |
| SynEcoSys database | — | Single-cell annotation database | <https://www.synecosys.com> |
| bedtools | — | Genomic feature annotation | <https://bedtools.readthedocs.io> |
| Integrative Genomics Viewer (IGV) | v2.4.x | Genomic visualization | <https://software.broadinstitute.org/software/igv/> |
| GEXSCOPE kits / Tissue Dissociation / Preservation | — | Sample preservation and dissociation | <https://www.singleronbio.com> |
| 10X Chromium Single Cell ATAC | — | Library construction and sequencing | <https://www.10xgenomics.com> |

**Footnote:**
“—” denotes cases where version information is not applicable, not available, or where the resource refers to an experimental kit or database rather than a software package. All listed URLs are official sources for the corresponding tools or databases.

**Supplementary Table S2. Summary of embryo and organ samples used in this study.**

| **No.** | **Tissues** | **Species** | **Single-cell RNA-Seq datasets** | **Single-cell ATAC-seq datasets** | **Weeks of gestation** |
| --- | --- | --- | --- | --- | --- |
| 1 | Embryo | Homo sapiens | Yes | Yes | 7 |
| 2 | Liver | Homo sapiens | Yes | No | 8 |
| 3 | Brain | Homo sapiens | Yes | Yes | 8 |
| 4 | Heart | Homo sapiens | Yes | No | 8 |
| 5 | Heart | Homo sapiens | Yes | No | 10 |
| 6 | Brain | Homo sapiens | Yes | Yes | 10 |
| 7 | Liver | Homo sapiens | Yes | Yes | 10 |
| 8 | Kidney | Homo sapiens | Yes | Yes | 10 |
| 9 | Liver | Homo sapiens | Yes | Yes | 11 |
| 10 | Brain | Homo sapiens | Yes | Yes | 11 |
| 11 | Kidney | Homo sapiens | Yes | Yes | 11 |
| 12 | Heart | Homo sapiens | Yes | No | 11 |
| 13 | Brain | Homo sapiens | Yes | No | 17 |
| 14 | Liver | Homo sapiens | Yes | No | 17 |
| 15 | Kidney | Homo sapiens | Yes | Yes | 17 |
| 16 | Heart | Homo sapiens | Yes | No | 17 |
| 17 | Spleen | Homo sapiens | Yes | Yes | 17 |

**Footnote**: All samples were obtained from spontaneous miscarriages with appropriate ethical approval. “Weeks of gestation (GW)” denotes the estimated gestational age at the time of collection. “Yes” indicates that sufficient viable cells were obtained and used for library preparation and sequencing; “No” indicates that scRNA-seq or scATAC-seq data were not generated for that sample. The term “Embryo” (GW07) refers to the whole embryo sample prior to organ dissection, whereas organ-specific samples (liver, brain, heart, kidney, spleen) were dissected at later gestational stages (GW08 - 17).

**Supplementary Table S3. Median gene and fragments counts per cell detected by** **scRNA-Seq and scATAC-Seq, respectively.**

| **Samples** | **Median genes per cell** | **Median fragments per cell** |
| --- | --- | --- |
| GW07 Embryo | 1619 | 13518 |
| GW08 Brain | 1011 | 46484 |
| GW08 Heart | 569 | NA |
| GW08 Liver | 1920 | NA |
| GW10 Brain | 928 | 10295 |
| GW10 Heart | 979 | NA |
| GW10 Kidney | 1738 | 4724 |
| GW10 Liver | 531 | 10226 |
| GW11 Brain | 787 | 21930 |
| GW11 Heart | 878 | NA |
| GW11 Kidney | 1451 | 27074 |
| GW11 Liver | 1238 | 19064 |
| GW17 Brain | 1050 | NA |
| GW17 Heart | 778 | NA |
| GW17 Kidney | 1814 | 8398 |
| GW17 Liver | 1786 | NA |
| GW17 spleen | 942 | 7294 |

**Footnote**: GW = gestational week. “Median genes per cell” indicates the median number of unique genes detected per cell by scRNA-seq after QC filtering. “Median fragments per cell” indicates the median number of unique fragments per cell detected by scATAC-seq. “NA” = not available, because scATAC-seq was not performed for that sample or cell numbers were below quality threshold.

**Supplementary Table S4. Cell-type abbreviations and representative gene markers used for cell annotation.**

| **Cell type** | **Abbreviation** | **Markers** |
| --- | --- | --- |
| Astrocytes | Astrocytes | SLC1A3, GLUL, GFAP |
| B cells | B cells | MS4A1, CD79A, CD79B |
| Brain endothelial cells | B-ECs | CDH5, PECAM1, CLDN5 |
| Cardiomyocytes | Cardiomyocytes | TNNI3, TNNT2, MYH6, NPPA, TNNC1 |
| Choroid plexus cells | CPCs | EPCAM, TTR, MSX1, KCNJ13 |
| Dendritic cells | DCs | CD1C, CD1E, HLA-DRB1, HLA-DPA1 |
| Distal tubule cells | DTCs | LIMCH1, MAL, SOX9 |
| Endothelial cells | ECs | CDH5, PECAM1, VWF, CLDN5, KDR |
| Endocrine cells | Endocrine cells | CHGA, SCG2, TH, CHGB |
| Epithelial cells | Epithelial cells | EPCAM, NPHS2, CUBN, KRT8, CLDN1 |
| Erythroblasts | Erythroblasts | AHSP, GYPA, HBA1 |
| Fibroblasts | Fibroblasts | DCN, LUM, COL1A2, COL1A1 |
| Granulocytes macrophages progenitor cells | GMP | PRTN3, MPO, AZU1 |
| Heart endothelial cells | H-ECs | CDH5, PECAM1, CLDN5, KDR, FABP4 |
| Hematocytes | Hematocytes | PTPRC, CD3D, MS4A1, LYZ, AHSP, GYPA, HBA1 |
| Hepatocytes | Hepatocytes | ALB, AFP, APOB |
| Haematopoietic stem cells | HSCs | SPINK2, SOX4, CD34, HOPX |
| Immature neurons | INs | NEUROD1, TBR1, DCX |
| Intermediate neural progenitors | INPs | EOMES, NEUROG2, SOX2 |
| Interneurons | Interneurons | DLX1, GAD1, GAD2 |
| Kidney endothelial cells | K-ECs | CDH5, PECAM1, EHD3, AQP1 |
| Liver endothelial cells | L-ECs | CDH5, CLEC4M, CLEC4G |
| Loop of Henle cells | LOH | UMOD, SLC12A1, CLDN16 |
| Macrophages | Macrophages | LYZ, MRC1, CD68, CD163, C1QC |
| Mast cells | Mast cells | TPSAB1, TPSB2, CPA3 |
| Megakaryocytes | Megakaryocytes | PF4, GP9, ITGA2B, PPBP |
| Mesoderm cells | MCs | FOXC1, MEST, MDK, PDGFRA, FOXC2 |
| Microglial cells | Microglial cells | P2RY12, CX3CR1 |
| Mesenchymal stem cells | MSCs | THY1, ALCAM, FGF7, MEIS1 |
| Mural cells | Mural cells | ACTA2, RGS5, TAGLN, KCNJ8 |
| Myoblasts | Myoblasts | ACTA1, MYOD, MYF6, SIX1 |
| Myocytes | Myocytes | TNNI3, TNNT2, MYH6, NPPA, TNNC1 |
| Neural cells | Neural cells | SNAP25, DCX, SYT1, STMN2 |
| Neural precursor cells | Neural precursor cells | SNAP25, DCX, SYT1, STMN2 |
| Neuroendocrine cells | Neuroendocrine cells | SST, CHGA, CHGB, NPY |
| Neutrophils | Neutrophils | LYZ, CSF3R, CXCR2, FCGR3B, CAMP, LCN2 |
| Nephron progenitor cells | NPCs | TMEM100, CITED1, PAX2, PAX8, LHX1 |
| Neural stem cells | NSCs | SOX2, SFRP1, PAX6, HES1, SOX9 |
| Oligodendrocyte precursor cells | OPCs | BCAN, PDGFRA, OLIG1 |
| Podocytes | Podocytes | MAFB, PODXL, PTPRO |
| Proximal tubule cells | PTCs | CUBN, SLC13A1, LRP2 |
| Retinal ganglion cells | RGCs | SNCG, POU4F1, NEFL, ISL1 |
| Schwann cells | Schwann cells | PLP1, LGI4, SOX2, MPZ |
| Spleen endothelial cells | S-ECs | CDH5, PECAM1, CLDN5, KDR |
| Steroid endocrine cells | SECs | STAR, CYP11A1, CYP17A1, CYP21A2 |
| Stromal cells | Stromal cells | DCN, LUM, VIM, COL5A1 |
| T cells | T cells | CD3D, CD3E, CD3G |
| Ureter epithelial cells | UECs | S100P, UPK1A |

**Footnote:** Marker genes were curated from published developmental and lineage studies and used for cell type annotation in scRNA-seq datasets. The listed genes represent canonical markers, and cell identities were assigned based on the combined expression of multiple markers rather than single genes. For overlapping categories (e.g., Neural precursor cells and Neural cells), assignment was determined by both marker expression level and developmental trajectory context. All abbreviations are consistent with those used throughout the main text and figure legends.

**Supplementary Table S5.** Cell counts detected by scRNA-Seq and scATAC-Seq

**Supplementary Table S5a.** The numbers of cells detected out in different samples using scRNA-Seq.

**Supplementary Table S5b.** The numbers of cells detected out in different samples using scATAC-Seq.

**Supplementary Table S5c.** The numbers of mesoderm cells detected out in different samples using scRNA-Seq.

**Supplementary Table S5d.** The numbers of mesoderm cells detected out in different samples using scATAC-Seq.

**Supplementary Table S5e.** The numbers of endothelial cells detected out in different samples using scRNA-Seq.
