# Supplementary material for: Molecular architecture of mesoderm cells across early to middle stage of human embryo development at single-cell resolution
Source: BMC Mol Cell Biol. 2025 Dec 25;27:3. doi: 10.1186/s12860-025-00561-9 (PMC12849250; doi:10.1186/s12860-025-00561-9)
Supplement: Supplementary file 3 — Supplementary Material 3 [file 12860_2025_561_MOESM3_ESM.docx]

**Supplementary Fig. S1. Expression of marker genes of major cell clusters and cell percentages across tissues and stages.** a, Violin plots showing the distribution of detected gene numbers (top) and unique molecular identifier (UMI) counts (bottom) across single cells from one whole embryo at GW07 and fetal organs (brain, heart, liver, kidney, spleen) at gestational weeks 8–17. UMIs represent transcript counts, providing a measure of sequencing depth per cell. Most cells displayed robust gene detection and consistent UMI levels, indicating high-quality single-cell transcriptomic profiles suitable for downstream analysis. b, Single-cell transcriptomic landscape of one entire embryo (GW07) and five fetal organs (brain, heart, liver, kidney, spleen) across gestational weeks 8–17. UMAP visualization shows six major cellular compartments (endocrine cells, myocytes, epithelial cells, neural cells, stromal cells, and hematocytes), and the bar plot on the right depicts their relative proportions across tissues and developmental stages. c-d, Violin plots showing the expression levels of representative cell type markers across six major cell types and 38 sub-clusters, as determined by scRNA-seq analysis. e, The percentage of 38 sub-clusters distributed in each sample at different stages of gestation and different tissues by scRNA-seq assay. f, UMAP visualization of cell types both detected out by scRNA-seq and scATAC-seq assays. DCs, Dendritic cells; DTCs, Distal tubule cells; ECs, Endothelial cells; HSCs, Haematopoietic stem cells; Imm neurons, Immature neurons; MCs, Mesoderm cells; MSCs, Mesenchymal stem cells; NPCs, Nephron progenitor cells; NSCs, Neural stem cells; OPCs, Oligodendrocyte precursor cells; PTCs, Proximal tubule cells; RGCs, Retinal ganglion cells; SECs, Steroid endocrine cells; UECs, Ureter epithelial cells.

**Supplementary Figure S2. Lineage inference and gene dynamics across fetal organs.** Single-cell developmental trajectories were reconstructed using Monocle 2 based on scRNA-seq data from one entire embryo (GW07) and fetal organs (brain, heart, liver) at gestational weeks 8–17. a, c, e, Trajectories of brain, heart, and liver cells, showing the developmental ordering of major cellular subtypes across different gestational stages together with their pooled trajectories (“all sample”). b, d, f, Heatmaps displaying dynamic expression patterns of the top 30 pseudotime-regulated genes within each organ-specific trajectory, identified by trajectory analysis using Monocle2, which models gene expression changes along inferred differentiation paths: b, neural cell lineages; d, heart cell lineages; f, liver cell lineages. Imm neurons, Immature neurons; Imm neurons, Immature neurons; INPs, Intermediate neural progenitors; NSCs, Neural stem cells; OPCs, Oligodendrocyte precursor cells; ECs, Endothelial cells; MCs, Mesoderm cells; GMPs, Granulocyte–monocyte progenitors; HSCs, Hematopoietic stem cells; MCs, Mesoderm cells.

**Supplementary Fig. S3. Molecular landscapes of MC sub-populations.** a, Average expression of canonical marker genes across the seven MC sub-clusters (MC1–MC7). Dot color indicates average scaled expression, while dot size indicates the percentage of cells within each cluster expressing the gene. Average expression refers to the normalized mean expression level of a given gene calculated across all cells within a specific cluster. b, Heatmap showing the top 10 TFs with the highest SCENIC activity scores (AUC values) in each MC sub-cluster (MC1–MC7). TFs are ranked within each cluster by activity. Numbers previously shown in parentheses (e.g., 32g) correspond to regulon IDs used by SCENIC/cisTarget for internal ranking. c, Expression dynamics of *EGR1* and its 28 target genes along the inferred pseudotime trajectory. Rows represent genes; values are row Z-scores (standardized within each gene across pseudo-time). d, Kyoto Encyclopedia of Genes and Genomes (KEGG) enrichment of the 28 genes targeted by *EGR1* in the seven MC sub-clusters. e, Joint UMAP embedding of single-cell transcriptomic (scRNA-seq, blue) and chromatin accessibility (scATAC-seq, red) profiles. Cells from both modalities exhibited largely overlapping distributions, confirming consistency between transcriptional states and underlying chromatin landscapes.

**Supplementary Fig. S4. Landscapes of cardiac cells.** a, Dot plot showing average expression of canonical marker genes across cardiac cell types. Dot color indicates scaled average expression; dot size indicates the percentage of cells in each population expressing the gene. Average expression refers to the normalized mean expression level of a given gene calculated across all cells within a specific cluster. Cell types include cardiomyocytes, ECs, endocardial cells, mural cells, fibroblasts, neuroendocrine cells, and Schwann cells. b, Pseudotime trajectories of MC sub-clusters (MC1–MC7) and endocardial cells (C5) in samples from different organs (embryo, brain, heart, kidney, liver, spleen). To highlight organ-specific developmental dynamics, panels are arranged by organ rather than gestational stage. Colored dots represent cells from individual sub-clusters; black lines indicate the inferred backbone trajectory by Monocle2. c, Heatmap showing the top 10 TFs with the highest SCENIC activity scores (AUC values) in MC1–MC7 and C5. Each row is a TF, and each column a cell cluster; values are row Z-scores. TFs are ranked by activity. Numbers previously shown in parentheses (e.g., 32g) correspond to regulon IDs used by SCENIC/cisTarget for internal ranking. d, Trajectory of MCs and C5 (top row), with expression dynamics of *EMCN* and *TMEM100* mapped onto the trajectory (bottom row). Color gradient indicates pseudotime (top) or normalized expression level (bottom). e, Violin plots showing expression of five RAD21-target genes (*EMCN, POSTN, ECSCR, HBB, RNASE1*) across MC sub-clusters and C5. Statistics: Wilcoxon rank-sum test with multiple-testing correction.

**Supplementary Fig. S5. Trajectory and functional heterogeneity of mesoderm-to-EC differentiation.** a, UMAP visualization of ECs captured from different tissues (brain, heart, kidney, liver, spleen, and 7-week embryo) and developmental stages, as analyzed by scRNA-Seq data. Each dot represents one single cell; colors denote tissue of origin. b, UMAP visualization showing expression of canonical marker genes across the six EC clusters, confirming cluster identity. c, Monocle2 trajectory analysis between MC sub-clusters (MC1–MC7) and EC clusters. Solid black lines mark the backbone trajectory. Colored dots denote individual cells, labeled by cluster identity. d, Violin plot showing the expression of *MEF2C* in MC7 and and EC sub-clusters. P-value was calculated with Wilcox likelihood-ratio test. *, p < 0.05; **, p < 0.01; ***, p < 0.001; ****, p < 0.0001. e, Gene Ontology (GO) analysis of *ANGPT2* and *COL4A2* targeted by MEF2C, showing significant enrichment in angiogenesis- and vasculature-related processes.

**Supplementary Fig. S6. Hematopoietic lineage differentiation from mesodermal progenitors.** a, CytoTRACE analysis of hematopoietic clusters (H1–H11), showing entropy values that reflect differentiation potential. Eleven major clusters were identified: mast cells (H1), granulocyte–macrophage progenitors (GMP, H2), T cells (H3), microglia cells (H4), dendritic cells (DCs, H5), neutrophils (H6), hematopoietic stem cells (HSCs, H7), megakaryocytes (H8), B cells (H9), macrophages (H10), and erythroblasts (H11). b, Expression dynamics of *HBZ* along the pseudotime trajectory of hematopoietic cells, with cluster identity indicated by color. X-axis represents pseudotime (arbitrary units inferred by Monocle2); Y-axis represents normalized expression levels. The solid curve denotes a smoothed expression trend. c, UMAP plots showing expression levels of *HBQ1, HBM,* and *HBG1* across hematopoietic sub-clusters. Each dot is a single cell; dot color indicates scaled expression. d, Schematic model summarizing heterogeneous MC differentiation, illustrating developmental progression toward heart cells, EC sub-clusters, and hematopoietic lineages including erythroblasts, mast cells, and neutrophils.
